# Supplementary material for: pGlycoQuant with a deep residual network for quantitative glycoproteomics at intact glycopeptide level
Source: Nat Commun. 2022 Dec 7;13:7539. doi: 10.1038/s41467-022-35172-x (PMC9729625; doi:10.1038/s41467-022-35172-x)
Supplement: Supplementary file 1 — Supplementary Information [file 41467_2022_35172_MOESM1_ESM.docx]

Supplementary Information of

pGlycoQuant with a deep residual network for quantitative glycoproteomics at intact glycopeptide level enabling the functional exploration of site-specific glycosylation

Siyuan Kong^#,1^, Pengyun Gong^#,2^, Wenfeng Zeng^#,3,4^, Biyun Jiang^#,1^, Xinhang Hou^2^, Yang Zhang^1^, Huanhuan Zhao^1^, Mingqi Liu^1^, Guoquan Yan^1^, Xinwen Zhou^1^, Xihua Qiao^2^, Mengxi Wu^1^, Pengyuan Yang ^1,5,6^, Chao Liu*^,2,3^, Weiqian Cao*^,1,5^

1. Shanghai Fifth People's Hospital and Institutes of Biomedical Sciences, Fudan University, Shanghai, China

2. School of Engineering Medicine & School of Biological Science and Medical Engineering, Beihang University, Beijing, China; and Key Laboratory of Big Data-Based Precision Medicine (Beihang University), Ministry of Industry and Information Technology

3. Key Lab of Intelligent Information Processing of Chinese Academy of Sciences (CAS), Institute of Computing Technology, CAS, Beijing, China

4. Proteomics and Signal Transduction, Max Planck Institute of Biochemistry, Martinsried, Germany

5. NHC Key Laboratory of Glycoconjugates Research, Fudan University, Shanghai, China

6. Deceased

# These authors contributed equally to this work

* To whom correspondence should be addressed:

W.C.([wqcao@fudan.edu.cn](mailto:wqcao@fudan.edu.cn)), C.L. ([liuchaobuaa@buaa.edu.cn](mailto:liuchaobuaa@buaa.edu.cn))

Table of contents

[Supplementary Table 1 Sources of Data. 4](#_Toc114841537)

[Supplementary Table 2 Sources of Software tools. 5](#_Toc114841538)

[Supplementary Table 3 Parameters of all software tools. 6](#_Toc114841539)

[Supplementary Table 4 Quantitation results of all software tools on the three benchmark datasets. 11](#_Toc114841540)

[Supplementary Figure 1 Workflow of pGlycoQuant. 14](#_Toc114841541)

[Supplementary Figure 2 Deep learning-based evidence matching model. 15](#_Toc114841542)

[Supplementary Figure 3 Matching Scoring distribution of pGlycoQuant. 16](#_Toc114841543)

[Supplementary Figure 4 The two-glycoproteome experimental design for the FQR estimation of pGlycoQuant with MBR. 17](#_Toc114841544)

[Supplementary Figure 5 The fold change-(de)glycoproteome experimental design for quantitative accuracy estimation of pGlycoQuant. 18](#_Toc114841545)

[Supplementary Figure 6 PMVT and PMVL. 20](#_Toc114841546)

[Supplementary Figure 7 An example of missing values from label-free data. 21](#_Toc114841547)

[Supplementary Figure 8 Comparison of the quantitative performance of all software tools on SILAC-labeled 293T cell data at glycopeptide level in the aspect of Pearson correlation and standard deviation. 22](#_Toc114841548)

[Supplementary Figure 9 Comparison of the quantitative performance of all software tools on label-free HeLa cell data at glycopeptide level in the aspect of Pearson correlation and standard deviation. 23](#_Toc114841549)

[Supplementary Figure 10 Comparison of the quantitative performance of all software tools on TMT-labeled 293T cell data at glycopeptide level in the aspect of Pearson correlation and standard deviation. 25](#_Toc114841550)

[Supplementary Figure 11 The quantitation results of the fold change-(de)glycoproteome data from IgG reported by different software tools. 26](#_Toc114841551)

[Supplementary Figure 12 The quantitation results of the fold change-(de)glycoproteome data from fission yeast reported by different software tools. 28](#_Toc114841552)

[Supplementary Figure 13 The quantitation results of the fold change-(de)glycoproteome data from human serum reported by different software tools. 30](#_Toc114841553)

[Supplementary Figure 14 The candidate glycan database constructed for MIR analysis. 32](#_Toc114841554)

[Supplementary Figure 15 The experimental design for validation of MIR using benchmarked N-glycopeptides. 33](#_Toc114841555)

[Supplementary Figure 16 The isotope distribution of the captured glycopeptide evidences in MS1 scans for the three abnormal score for 1SA-GPs in Figure 3f. 34](#_Toc114841556)

[Supplementary Figure 17 MIR analysis of N-glycopeptides in human IgG. 35](#_Toc114841557)

[Supplementary Figure 18 High repeatability of the proteome quantitative results among three cell lines 36](#_Toc114841558)

[Supplementary Figure 19 High repeatability of the intact glycopeptide quantitative results among three cell lines 37](#_Toc114841559)

[Supplementary Figure 20 Gene ontology (GO) analyses of proteome 38](#_Toc114841560)

[Supplementary Figure 21 Gene ontology (GO) analyses of glycoproteome 40](#_Toc114841561)

[Supplementary Figure 22 Distribution of glycan size and glycan type in three HCC cell lines 41](#_Toc114841562)

[Supplementary Figure 23 Differential expressed proteins of glycan-related genes in three HCC cell lines 42](#_Toc114841563)

[Supplementary Figure 24 Site-specific glycosylation level of L1CAM in three HCC cell lines after normalization within a cell line or among three cell lines. 43](#_Toc114841564)

[Supplementary Figure 25 A comparison of differential intact glycopeptides without and with normalization to protein abundance. 44](#_Toc114841565)

[Supplementary Figure 26 Silencing L1CAM inhibited migration and invasion ability of LM3 cells 46](#_Toc114841566)

[Supplementary Note 1 Experimental methods for the two-glycoproteome data obtaining. 47](#_Toc114841567)

[Supplementary Note 2 Experimental methods for the fold change-(de)glycoproteome data obtaining. 49](#_Toc114841568)

[Supplementary Note 3 Sample preparation and LC-MS/MS Methods for the three benchmark datasets and HCC cell lines. 50](#_Toc114841569)

[Supplementary Note 4 Methods for invitro molecular biology experiments. 55](#_Toc114841570)

# Supplementary Table 1 Sources of Data.

| **Sample dataset** | **Data** | **Sample source** | **The type of raw data** | **The number of raw data** |
| --- | --- | --- | --- | --- |
| The two-glycoproteome dataset | Human serum data | Human serum | label free | 3 |
|  | Fission yeast data | Fission yeast | label free | 3 |
| The fold change-(de)glycoproteome dataset | Human IgG glycopeptide data | Human IgG | label free | 6 |
|  | Human IgG deglycopeptide data | Human IgG | label free | 6 |
|  | Fission yeast glycopeptide data | Fission yeast | label free | 6 |
|  | Fission yeast deglycopeptide data | Fission yeast | label free | 6 |
|  | Human serum glycopeptide data | Human serum | label free | 6 |
|  | Human serum deglycopeptide data | Human serum | label free | 6 |
| Three benchmark datasets | Label-free HeLa cell data | HeLa cell | label free | 9 |
|  | SILAC-labeled 293T cell data | 293T cell | SILAC | 3 |
|  | TMT-labeled 293T cell data | 293T cell | TMT labeling | 3 |

# Supplementary Table 2 Sources of Software tools.

| **Software portfolio** | **Identification software** | **Version of identification software** | **Quantification software** | **Version of quantitation software** |
| --- | --- | --- | --- | --- |
| Byonic+Byologic | Byonic | V4.0.1 | Byologic | V4.0.1 |
| Byonic+PD | Byonic | V2.16.11 | PD | 3.0 |
| Byonic+pGlycoQuant | Byonic | V4.0.1 | pGlycoQuant | v1.1_build20220920 |
| MSFragger-Glyco+ MSFragger-Glyco | MSFragger-Glyco | FragPipe15.0, MSFragger_3.2, philosopher_v3.3.12 (SILAC-labeled 293T cell data and label-free HeLa cell data) | MSFragger-Glyco | FragPipe15.0, MSFragger_3.2, philosopher_v3.3.12 (SILAC-labeled 293T cell data and label-free HeLa cell data) |
|  |  | FragPipe16.0, MSFragger_3.3, philosopher_v4.0.0  (TMT-labeled 293T cell data) |  | FragPipe16.0, MSFragger_3.3, philosopher_v4.0.0  (TMT-labeled 293T cell data) |
| MSFragger-Glyco+  pGlycoQuant | MSFragger-Glyco | FragPipe15.0, MSFragger_3.2, philosopher_v3.3.12 (SILAC-labeled 293T cell data and label-free HeLa cell data) | pGlycoQuant | v1.1_build20220920 |
|  |  | FragPipe16.0, MSFragger_3.3, philosopher_v4.0.0  (TMT-labeled 293T cell data) |  |  |
| pGlyco3+Skyline | pGlyco3 | pGlyco3 | Skyline | 21.2.1.455 |
| pGlyco3+  pGlycoQuant | pGlyco3 | pGlyco3 | pGlycoQuant | v1.1_build20220920 |

# Supplementary Table 3 Parameters of all software tools.

3-1 The two-glycoproteome dataset

| **Dataset** |  | Human serum data | | Fission yeast data |
| --- | --- | --- | --- | --- |
| **Software** | Analysis software | pGlyco3+pGlycoQuant | | |
| **Identification** | Identification software | pGlyco3 | | |
|  | Protein database | *H. sapiens* downloaded from UniProt (August 2018, 20,386 entries) | *S.pombe* downloaded from UniProt (August 2018, 5,273 entries) | |
|  | Digestion | Trypsin | | |
|  | Max miss cleavage | 2 | | |
|  | Fixed modification | Carbamidomethyl on Cys | | |
|  | Variable modification | Acetyl on Protein N-terminal; Oxidation on Met | | |
|  | Mode | N-glycan | | |
|  | Glyco database | pGlyco3 build-in human N-glycan database | | pGlyco3 build-in yeast N-glycan database |
|  | GPSM filter | 1% FDR | | |
|  | Fragmentation method | HCD | | |
|  | Precursor tolerance | 4ppm | | |
|  | Fragment tolerance | 20ppm | | |
| **Quantification** | Quantification software | pGlycoQuant | | |
|  | Quant type | label free | | |
|  | QC | 1% FQR | | |
|  | Quant level | MS1 | | |
|  | Precursor tolerance | 20ppm | | |
|  | Fragment tolerance | — | | |
|  | RT tolerance window | 2min | | |

3-2 The fold change-(de)glycoproteome dataset

| **Dataset** |  | Human IgG glycopeptide data | Human IgG deglycopeptide data | Fission yeast glycopeptide data | Fission yeast deglycopeptide data | Human serum glycopeptide data | Human serum  deglycopeptide data |
| --- | --- | --- | --- | --- | --- | --- | --- |
| **Software** | Analysis software | pGlyco3+pGlycoQuant | | | | | |
| **Identification** | Identification software | pGlyco3 | — | pGlyco3 | — | pGlyco3 | — |
|  | Protein database | Human IgG  downloaded from UniProt (August 2018) | — | *S.pombe*  downloaded from UniProt (August 2018, 5,273 entries) | — | *H. sapiens*  downloaded from UniProt (August 2018, 20,386 entries) | — |
|  | Digestion | Trypsin | — | Trypsin | — | Trypsin | — |
|  | Max miss cleavage | 2 | — | 2 | — | 2 | — |
|  | Fixed modification | Carbamidomethyl on Cys | — | Carbamidomethyl on Cys | — | Carbamidomethyl on Cys | — |
|  | Variable modification | Acetyl on Protein N-terminal; Oxidation on Met | — | Acetyl on Protein N-terminal; Oxidation on Met | — | Acetyl on Protein N-terminal; Oxidation on Met | — |
|  | Mode | N-glycan | — | N-glycan | — | N-glycan | — |
|  | Glycan database | pGlyco3 build-in human N-glycan database | — | pGlyco3 build-in yeast N-glycan database | — | pGlyco3 build-in human N-glycan database | — |
|  | GPSM filter | 1% FDR | — | 1% FDR | — | 1% FDR | — |
|  | Fragmentation method | HCD | — | HCD | — | HCD | — |
|  | Precursor tolerance | 4ppm | — | 4ppm | — | 4ppm | — |
|  | Fragment tolerance | 20ppm | — | 20ppm | — | 20ppm | — |
| **Quantification** | Quantification software | pGlycoQuant | | | | | |
|  | Quant type | label free | | | | | |
|  | QC | 1% FQR | | | | | |
|  | Quant level | MS1 | | | | | |
|  | Precursor tolerance | 20ppm | | | | | |
|  | Fragment tolerance | — | | | | | |
|  | RT tolerance window | 2min | | | | | |

3-3 Label-free HeLa cell data

| **Dataset** | Label-free HeLa cell data | | | | | | | | | |
| --- | --- | --- | --- | --- | --- | --- | --- | --- | --- | --- |
| **Software** | Analysis software | Byonic+  Byologic | | Byonic+  PD | Byonic+  pGlycoQuant | MSFragger-Glyco+  MSFragger-Glyco | MSFragger-Glyco+  pGlycoQuant | pGlyco3+  Skyline | | pGlyco3+  pGlycoQuant |
| **Identification** | Identification software | Byonic | | | | MSFragger-Glyco | | pGlyco3 | | |
|  | Protein database | *H. sapiens* downloaded from UniProt (August 2018, 20,386 entries) | | | | | | | | |
|  | Digestion | Trypsin | | | | Trypsin | | Trypsin | | |
|  | Max miss cleavage | 3 | | | | 2 | | 2 | | |
|  | Fixed modification | Carbamidomethyl on Cys | | | | Carbamidomethyl on Cys | | Carbamidomethyl on Cys | | |
|  | Variable modification | Acetyl on Protein N-terminal; Oxidation on Met | | | | Acetyl on Protein N-terminal; Oxidation on Met | | Acetyl on Protein N-terminal; Oxidation on Met | | |
|  | Mode | N-glycan | | | | N-Glycan | | N-glycan | | |
|  | Glyco database | 182 glycan compositions | | | | 182 glycan compositions | | pGlyco3 build-in human N-glycan database | | |
|  | GPSM filter | Score≥200&\|logProb\|≥2 | | | | 1% FDR | | 1% FDR | | |
|  | Fragmentation method | HCD | | | | HCD | | HCD | | |
|  | Precursor tolerance | 4ppm | | | | 20ppm | | 4ppm | | |
|  | Fragment tolerance | 20ppm | | | | 20ppm | | 20ppm | | |
| **Quantification** | Quantification software | Byologic | PD | | pGlycoQuant | MSFragger-Glyco | pGlycoQuant | Skyline | pGlycoQuant | |
|  | Quant type | label free | | | | | | | | |
|  | QC | — | | — | 1% FQR | 1% ion FDR | 1% FQR | — | 1% FQR | |
|  | Quant level | MS1 | | | | | | | | |
|  | Precursor tolerance | — | | 10ppm | 20ppm | 10ppm | 20ppm | — | 20ppm | |
|  | Fragment tolerance | — | | | | | | | | |
|  | RT tolerance window | — | | 10min | 2min | — | 2min | 2min | 2min | |

3-4 SILAC-labeled 293T cell data

| **Dataset** | SILAC-labeled 293T cell data | | | | | | | |
| --- | --- | --- | --- | --- | --- | --- | --- | --- |
| **Software** | Analysis software | Byonic+  Byologic | Byonic+  PD | Byonic+  pGlycoQuant | MSFragger-Glyco+  MSFragger-Glyco | MSFragger-Glyco+  pGlycoQuant | pGlyco3+  Skyline | pGlyco3+  pGlycoQuant |
| **Identification** | Identification software | Byonic | | | MSFragger-Glyco | | pGlyco3 | |
|  | Protein database | *H. sapiens* downloaded from UniProt (August 2018, 20,386 entries) | | | | | | |
|  | Digestion | Trypsin | | | Trypsin | | Trypsin | |
|  | Max miss cleavage | 3 | | | 2 | | 2 | |
|  | Fixed modification | K0R0: Carbamidomethyl on Cys | | | K0R0: Carbamidomethyl on Cys | | K0R0: Carbamidomethyl on Cys | |
|  |  | K6R6: Carbamidomethyl on Cys; Label 13C(6) on Lys and Arg | | | K6R6: Carbamidomethyl on Cys; Label 13C(6) on Lys and Arg | | K6R6: Carbamidomethyl on Cys; Label 13C(6) on Lys and Arg | |
|  | Variable modification | Acetyl on Protein N-terminal; Oxidation on Met | | | Acetyl on Protein N-terminal; Oxidation on Met | | Acetyl on Protein N-terminal; Oxidation on Met | |
|  | Mode | N-glycan | | | N-glycan | | N-Glycan | |
|  | Glycan database | 182 glycan compositions | | | 182 glycan compositions | | pGlyco3 build-in human N-glycan database | |
|  | GPSM filter | Score≥200&\|logProb\|≥2 | | | 1% FDR | | 1% FDR | |
|  | Fragmentation method | HCD | | | HCD | | HCD | |
|  | Precursor tolerance | 4ppm | | | 20ppm | | 4ppm | |
|  | Fragment tolerance | 20ppm | | | 20ppm | | 20ppm | |
| **Quantification** | Quantification software | Byologic | PD | pGlycoQuant | MSFragger-Glyco | pGlycoQuant | Skyline | pGlycoQuant |
|  | Quant type | SILAC | | | | | | |
|  | Quant level | MS1 | | | | | | |
|  | Precursor tolerance | — | 10ppm | 20ppm | 10ppm | 20ppm | — | 20ppm |
|  | Fragment tolerance | — | | | | | | |
|  | RT tolerance window | — | 10min | 2min | — | 2min | 2min | 2min |

3-5 TMT-labeled 293T cell data

| **Dataset** | TMT-labeled 293T cell data | | | | | | | |
| --- | --- | --- | --- | --- | --- | --- | --- | --- |
| **Software** | Analysis software | Byonic+  Byologic | Byonic+  PD | Byonic+  pGlycoQuant | MSFragger-Glyco+  MSFragger-Glyco | MSFragger-Glyco+  pGlycoQuant | pGlyco3+  Skyline | pGlyco3+  pGlycoQuant |
| **Identification** | Identification software | Byonic | | | MSFragger-Glyco | | pGlyco3 | |
|  | Protein database | *H. sapiens* downloaded from UniProt (August 2018, 20,386 entries) | | | | | | |
|  | Digestion | Trypsin | | | Trypsin | | Trypsin | |
|  | Max miss cleavage | 3 | | | 2 | | 2 | |
|  | Fixed modification | Carbamidomethyl on Cys; TMT6plex on Any N-terminal; TMT6plex on Lys | | | Carbamidomethyl on Cys; TMT6plex on Any N-terminal; TMT6plex on Lys | | Carbamidomethyl on Cys; TMT6plex on Any N-terminal; TMT6plex on Lys | |
|  | Variable modification | Acetyl on Protein N-Term; Oxidation on Met | | | Acetyl on Protein N-Term; Oxidation on Met | | Acetyl on Protein N-Term; Oxidation on Met | |
|  | Mode | N-glycan | | | N-glycan | | N-Glycan | |
|  | Glyco database | 182 glycan compositions | | | 182 glycan compositions | | pGlyco3 build-in human N-glycan database | |
|  | GPSM filter | Score≥200&\|logProb\|≥2 | | | 1% FDR | | 1% FDR | |
|  | Fragmentation method | HCD | | | HCD | | HCD | |
|  | Precursor tolerance | 4ppm | | | 20ppm | | 4ppm | |
|  | Fragment tolerance | 20ppm | | | 20ppm | | 20ppm | |
| **Quantification** | Quantification software | Byologic | PD | pGlycoQuant | MSFragger-Glyco | pGlycoQuant | Skyline | pGlycoQuant |
|  | Quant type | TMT | | | | | | |
|  | Quant level | — | MS2 | MS2 | MS2 | MS2 | MS2 | MS2 |
|  | Precursor tolerance | — | | | | | | |
|  | Fragment tolerance | — | 20ppm | 20ppm | — | 20ppm | — | 20ppm |
|  | RT tolerance window | — | | | | | | |

# Supplementary Table 4 Quantitation results of all software tools on the three benchmark datasets.

4-1 Label-free HeLa cell data

| **Dataset** | Label-free HeLa cell data | | | | | | |
| --- | --- | --- | --- | --- | --- | --- | --- |
| **Identification** | Byonic | Byonic | Byonic | MSFragger-Glyco | MSFragger-Glyco | pGlyco3 | pGylco3 |
| **Quantitation** | Byologic | PD | pGlycoQuant | MSFragger-Glyco | pGlycoQuant | Skyline | pGlycoQuant |
| **Number of GPSMs** | N/A | N/A | 31491 | N/A | 7849 | N/A | 26288 |
| **PMVL (%)** | N/A | N/A | 13.55% | N/A | 13.98% | N/A | 11.33% |
| **PMVT (%)** | N/A | N/A | 4.07% | N/A | 3.57% | N/A | 2.66% |
| **Pearson** | N/A | N/A | 0.983-0.995 | N/A | 0.984-0.994 | N/A | 0.986-0.995 |
| **Standard deviation** | N/A | N/A | 0.129-0.308 | N/A | 0.136-0.298 | N/A | 0.124-0.296 |
| **Number of glycopeptides*** | 2716 | 2564 | 2740 | 607* | 1651 | 1931/2348^#^ | 2348 |
| **PMVL (%)** | 61.45% | 10.76% | 8.72% | 27.68% | 8.96% | 18.19% | 6.94% |
| **PMVT (%)** | 30.12% | 4.89% | 3.42% | 13.45% | 2.43% | 17.81% | 2.03% |
| **Pearson** | 0.941-0.973 | 0.955-0.980 | 0.974-0.995 | 0.940-0.987 | 0.976-0.994 | 0.877-0.961 | 0.979-0.996 |
| **Standard deviation** | 0.186-0.383 | 0.212-0.372 | 0.179-0.386 | 0.159-0.393 | 0.166-0.334 | 0.231-0.407 | 0.172-0.359 |
| **Number of glycoproteins** | N/A | N/A | 405 | 333 | 362 | N/A | 483 |
| **PMVL (%)** | N/A | N/A | 7.41% | 37.24% | 9.12% | N/A | 9.73% |
| **PMVT (%)** | N/A | N/A | 3.95% | 27.83% | 2.92% | N/A | 2.48% |
| **Pearson** | N/A | N/A | 0.983-0.998 | 0.971-0.998 | 0.986-0.997 | N/A | 0.991-0.998 |
| **Standard deviation** | N/A | N/A | 0.120-0.301 | 0.068-0.244 | 0.121-0.297 | N/A | 0.123-0.304 |

* MSFragger-Glyco reports quantitation results of glycopeptides at peptide sequence level, other software report at modified glycopeptide level.

^#^ The number after the slash indicates the number of input entries for quantitation by Skyline, the number before the slash indicates the number of quantitation results reported by Skyline.

The N/A indicates the value is not reported by the software.

4-2 SILAC-labeled 293T cell data

| **Dataset** | SILAC-labeled 293T cell data | | | | | | |
| --- | --- | --- | --- | --- | --- | --- | --- |
| **Identification** | Byonic | Byonic | Byonic | MSFragger-Glyco | MSFragger-Glyco | pGlyco3 | pGylco3 |
| **Quantitation** | Byologic | PD | pGlycoQuant | MSFragger-Glyco | pGlycoQuant | Skyline | pGlycoQuant |
| **Number of GPSMs** | N/A | N/A | 5389 | N/A | 1553 | N/A | 4492 |
| **PMVL (%)** | N/A | N/A | 1.15% | N/A | 2.51% | N/A | 0.31% |
| **PMVT (%)** | N/A | N/A | 0.68% | N/A | 1.26% | N/A | 0.16% |
| **Pearson** | N/A | N/A | 0.830 | N/A | 0.865 | N/A | 0.831 |
| **Standard deviation** | N/A | N/A | 0.982 | N/A | 1.001 | N/A | 0.940 |
| **Number of glycopeptides** | 985 | 627 | 1021 | 361* | 523 | 807/827^#^ | 827 |
| **PMVL (%)** | 53.40% | 20.89% | 1.47% | 11.08% | 0.57% | 2.54% | 0.73% |
| **PMVT (%)** | 26.75% | 13.56% | 0.93% | N/A | 0.29% | 2.48% | 0.36% |
| **Pearson** | 0.665 | 0.793 | 0.879 | N/A | 0.892 | 0.649 | 0.863 |
| **Standard deviation** | 1.693 | 0.957 | 1.077 | 0.975 | 1.042 | 1.037 | 1.11 |
| **Number of glycoproteins** | N/A | N/A | 253 | 148 | 181 | N/A | 252 |
| **PMVL (%)** | N/A | N/A | 1.98% | 4.73% | 0.55% | N/A | 1.19% |
| **PMVT (%)** | N/A | N/A | 1.19% | N/A | 0.28% | N/A | 0.60% |
| **Pearson** | N/A | N/A | 0.878 | N/A | 0.92 | N/A | 0.882 |
| **Standard deviation** | N/A | N/A | 0.945 | 0.769 | 0.735 | N/A | 1.080 |

* MSFragger-Glyco reports quantitation results of glycopeptides at peptide sequence level, other software report at modified glycopeptide level.

^#^ The number after the slash indicates the number of input entries for quantitation by Skyline, the number before the slash indicates the number of quantitation results reported by Skyline.

The N/A indicates the value is not reported by the software. MSFragger-Glyco reports the ratios of light/heave intensity instead of intensity values, thus the PMVL and PMVT values and Pearson correlation coefficient cannot be calculated.

4-3 TMT-labeled 293T cell data

| **Dataset** | TMT-labeled 293T cell data | | | | | | |
| --- | --- | --- | --- | --- | --- | --- | --- |
| **Identification** | Byonic | Byonic | Byonic | MSFragger-Glyco | MSFragger-Glyco | pGlyco3 | pGylco3 |
| **Quantitation** | Byologic | PD | pGlycoQuant | MSFragger-Glyco | pGlycoQuant | Skyline | pGlycoQuant |
| **Number of GPSMs** | N/A | N/A | 857 | 1463 | 1461 | N/A | 1370 |
| **PMVL (%)** | N/A | N/A | 0.82% | 0.68% | 0.75% | N/A | 0.88% |
| **PMVT (%)** | N/A | N/A | 0.76% | 0.55% | 0.58% | N/A | 0.69% |
| **Pearson** | N/A | N/A | 0.983 | 0.983 | 0.983 | N/A | 0.972 |
| **Standard deviation** | N/A | N/A | 0.133 | 0.127 | 0.127 | N/A | 0.123 |
| **Number of glycopeptides** | N/A | 322 | 372 | 332* | 670 | 630/636^#^ | 636 |
| **PMVL (%)** | N/A | 31.99% | 0.27% | 4.52% | 0.15% | 11.64% | 0.16% |
| **PMVT (%)** | N/A | 31.99% | 0.27% | 4.52% | 0.07% | 11.64% | 0.16% |
| **Pearson** | N/A | 0.977 | 0.995 | 0.999 | 0.996 | 0.997 | 0.990 |
| **Standard deviation** | N/A | 0.112 | 0.105 | 0.078 | 0.099 | 0.068 | 0.094 |
| **Number of glycoproteins** | N/A | N/A | 117 | 205 | 212 | N/A | 169 |
| **PMVL (%)** | N/A | N/A | 0.00% | 3.90% | 0.00% | N/A | 0.00% |
| **PMVT (%)** | N/A | N/A | 0.00% | 3.90% | 0.00% | N/A | 0.00% |
| **Pearson** | N/A | N/A | 0.998 | 0.999 | 0.999 | N/A | 0.999 |
| **Standard deviation** | N/A | N/A | 0.092 | 0.07 | 0.070 | N/A | 0.063 |

* MSFragger-Glyco reports quantitation results of glycopeptides at peptide sequence level, other software report at modified glycopeptide level.

^#^ The number after the slash indicates the number of input entries for quantitation by Skyline, the number before the slash indicates the number of quantitation results reported by Skyline.

The N/A indicates the value is not reported by the software.

# Supplementary Figure 1 Workflow of pGlycoQuant.

**
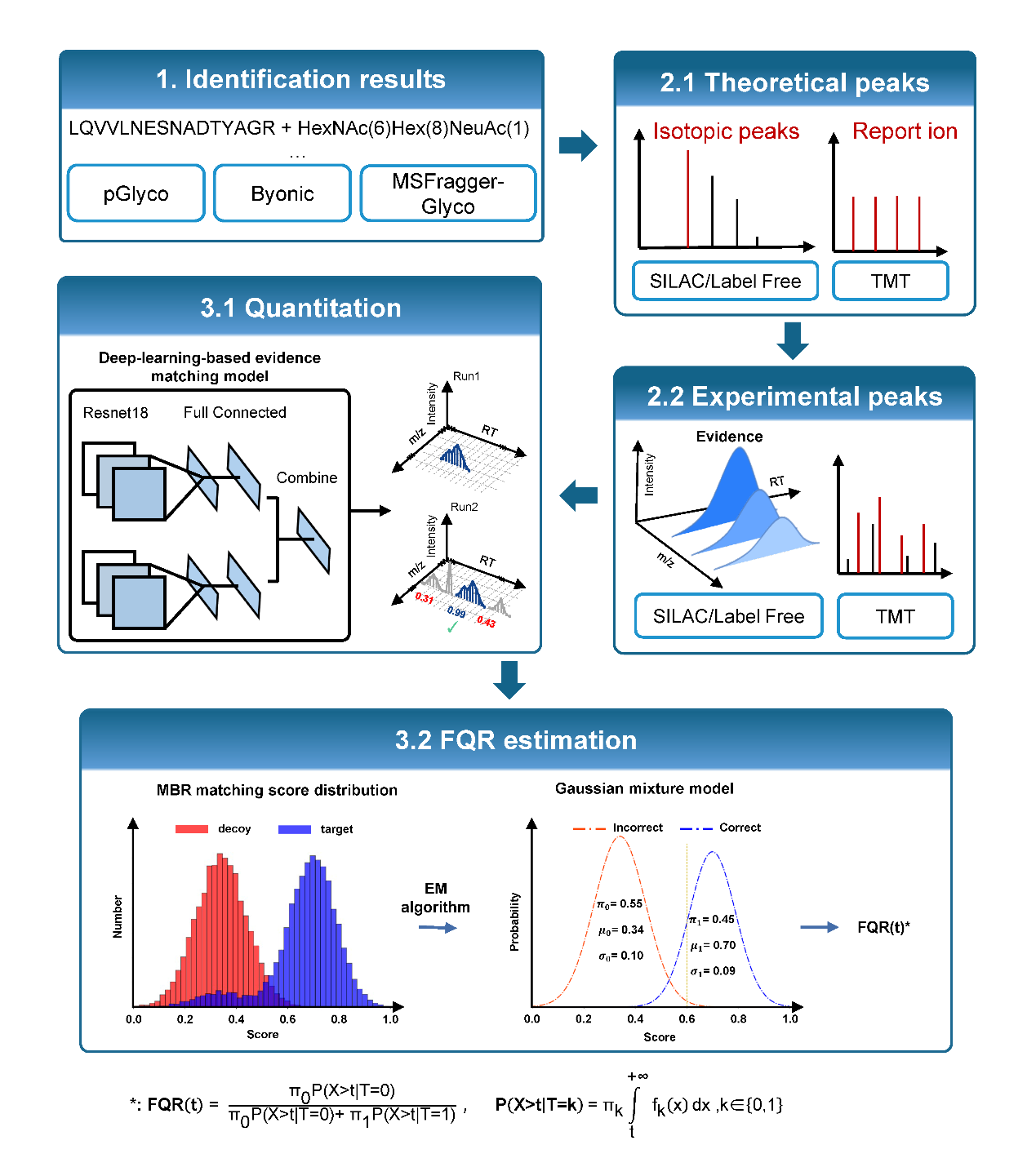
**The workflow of pGlycoQuant consists of three steps: first reading the identification results from search engines, then extracting the signals, and finally processing the quantitation results.

# Supplementary Figure 2 Deep learning-based evidence matching model.

**
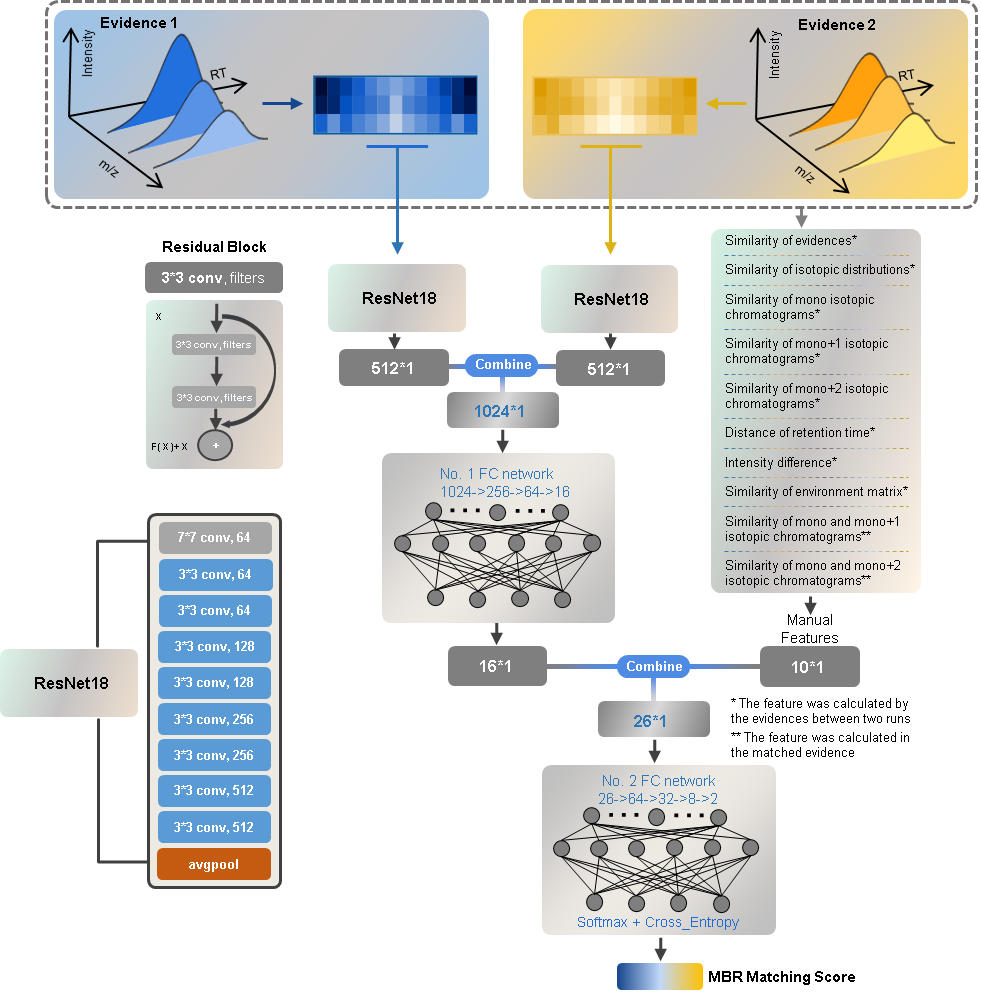
**We consider the evidence as a matrix, similar to a picture in computational vision, so ResNet18 could be used to transform the matrix to a vector. Two fully connected (FC) networks are used to calculate the matching score of the given two evidences.

# Supplementary Figure 3 Matching Scoring distribution of pGlycoQuant.


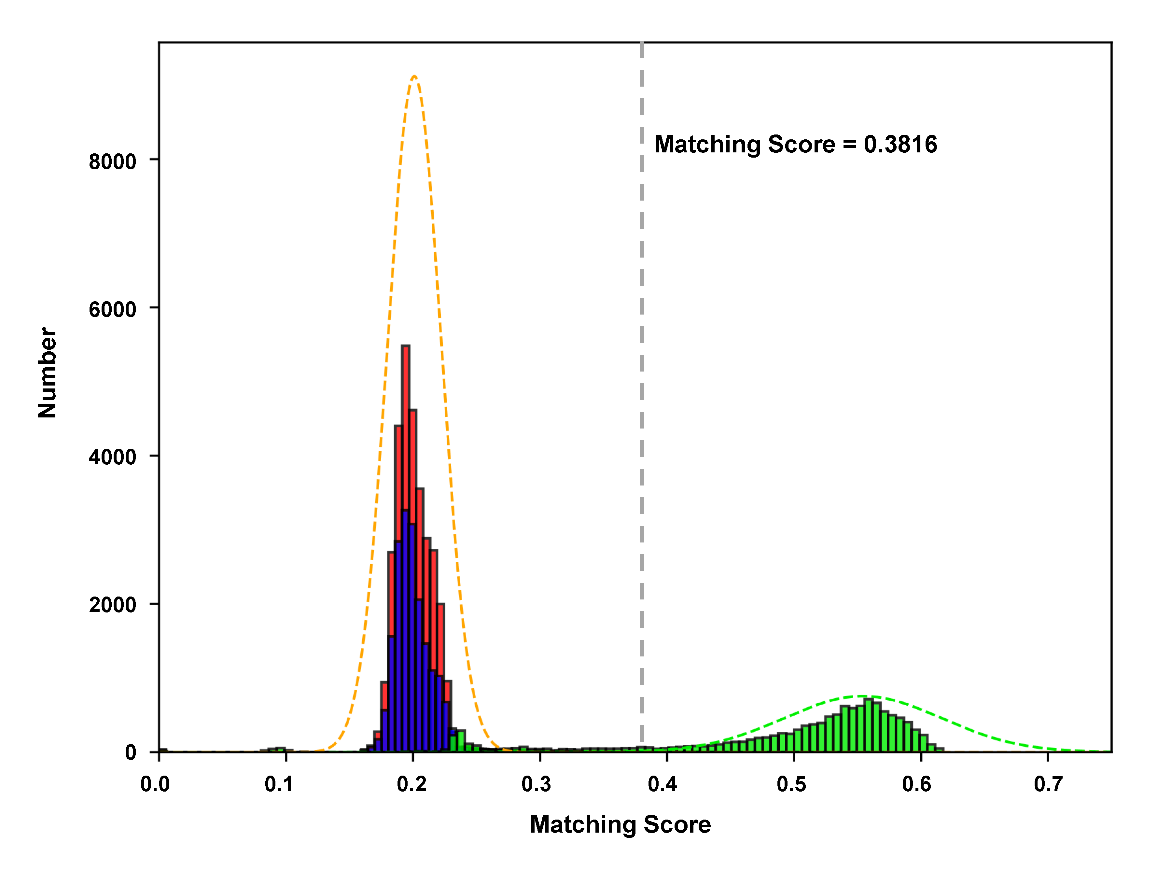
We used the quantitative results of the two-glycoproteome data to illustrate matching scoring distribution of pGlycoQuant. The red bars represent quantitative results from the decoys, which are considered to be false positive samples. The blue bars represent the quantitative results of serum glycopeptides in yeast data and yeast glycopeptides in serum data by MBR algorithm from the target library, which are considered to be false positive samples generated by the entrapment strategy. The green bars represent the quantitative results of serum glycopeptides in serum data and yeast glycopeptides in yeast data by MBR algorithm from the target library, which is considered to be a mixture of true and false positive samples. The orange and the green dashed line represent the fitted mixture Gaussian distribution with all matching scores. The grey dotted line represents the scoring threshold to distinguish true and false positive results under the filter condition of FQR<=0.01, and the value is 0.3816.


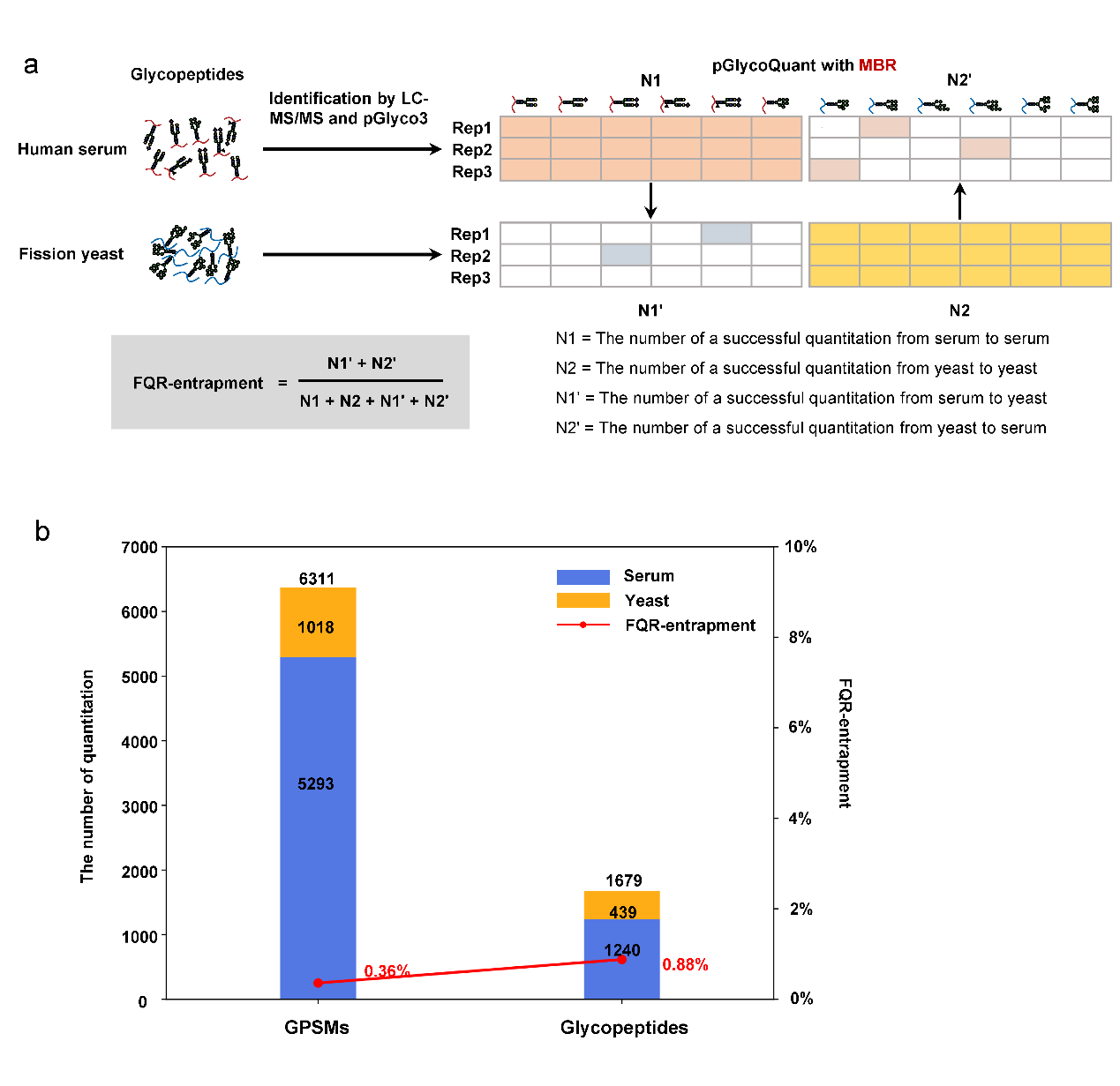
Supplementary Figure 4 The two-glycoproteome experimental design for the FQR estimation of pGlycoQuant with MBR. (a) The two-glycoproteome experimental design for entrapment-based false quantitative rate (FQR-entrapment) calculation. (b) The quantitation results of the two-glycoproteome data with entrapment-based FQR calculation. The bar indicates the number of quantitation results of GPSMs and glycopeptides in human serum (blue bar) and fission yeast (orange bar), respectively. The point plot indicates the FQR-entrapment calculated based the equation in figure a.

Supplementary Figure 5 The fold change-(de)glycoproteome experimental design for quantitative accuracy estimation of pGlycoQuant. (a) The fold change-(de)glycoproteome experimental workflow. Glycopeptides enriched from standard glycoprotein IgG/fission yeast/ human serum, were divided into four conditions, respectively: 1) 1 μg glycopeptides without treatment, 2) 1 μg glycopeptides treated with PNGase F (deglycopeptides), 3) 200 ng glycopeptides without treatment, 4) 200 ng glycopeptides treated with PNGase F (deglycopeptides). Samples from each condition were analyzed by LC-MS/MS with triplicates and identified by pGlyco3. Then, quantitation with MBR was performed among conditions. Histogram with fitting for visualization of the quantitative ratio between condition 1 and condition 3 in IgG (b), fission yeast (c), and human serum samples (d). Histogram with fitting for visualization of quantitative ratio between deglycopeptide condition and glycopeptide condition in IgG (e), fission yeast (f), and human serum samples(g). The rare glycopeptides quantified in the deglycoproteome experiment for fission yeast and human serum are probably due to the non-100% efficiency of PNGase F on removal of glycans in complex samples. The original quantitation data can be obtained from the MassIVE (https://massive.ucsd.edu/) with identifier MSV000089484 (see the Data availability in the manuscript for the detailed download information).


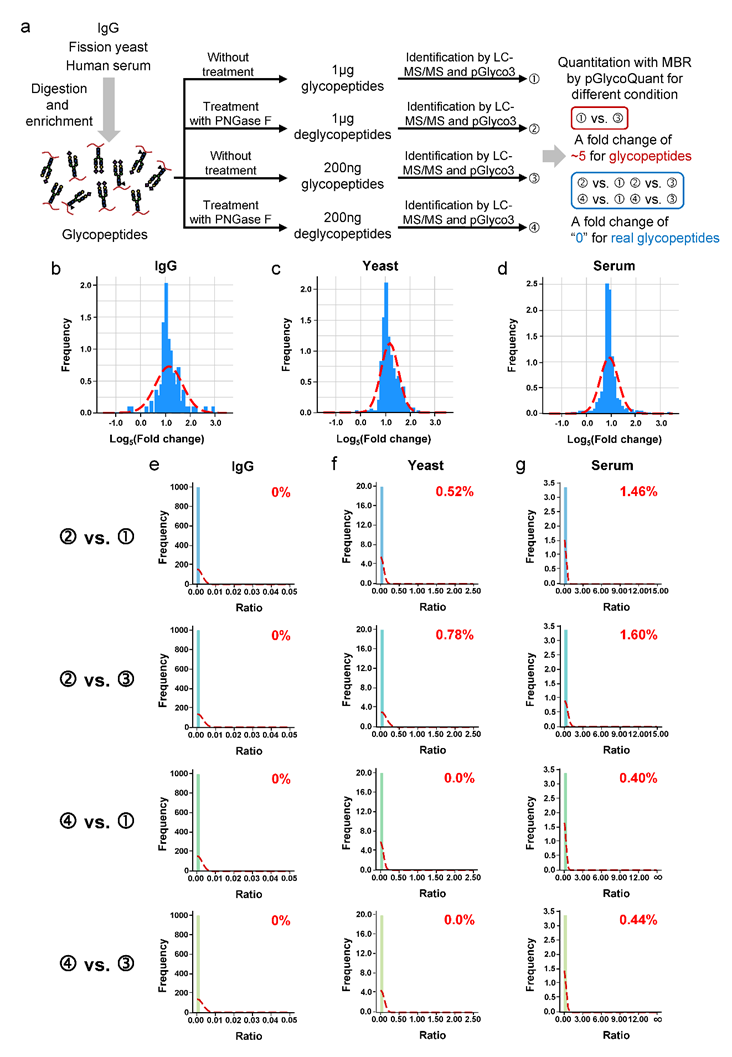


# Supplementary Figure 6 PMVT and PMVL.

We define two indicators, proportion of missing value in line (PMVL) and proportion of missing value in total (PMVT). PMVL (%) is the number of quantified IDs (GPSMs/glycopeptides/ proteins) with more than one missing value divided by the number of all quantified IDs. PMVT (%) is the number of individual missing values divided by the number of all quantitation values.


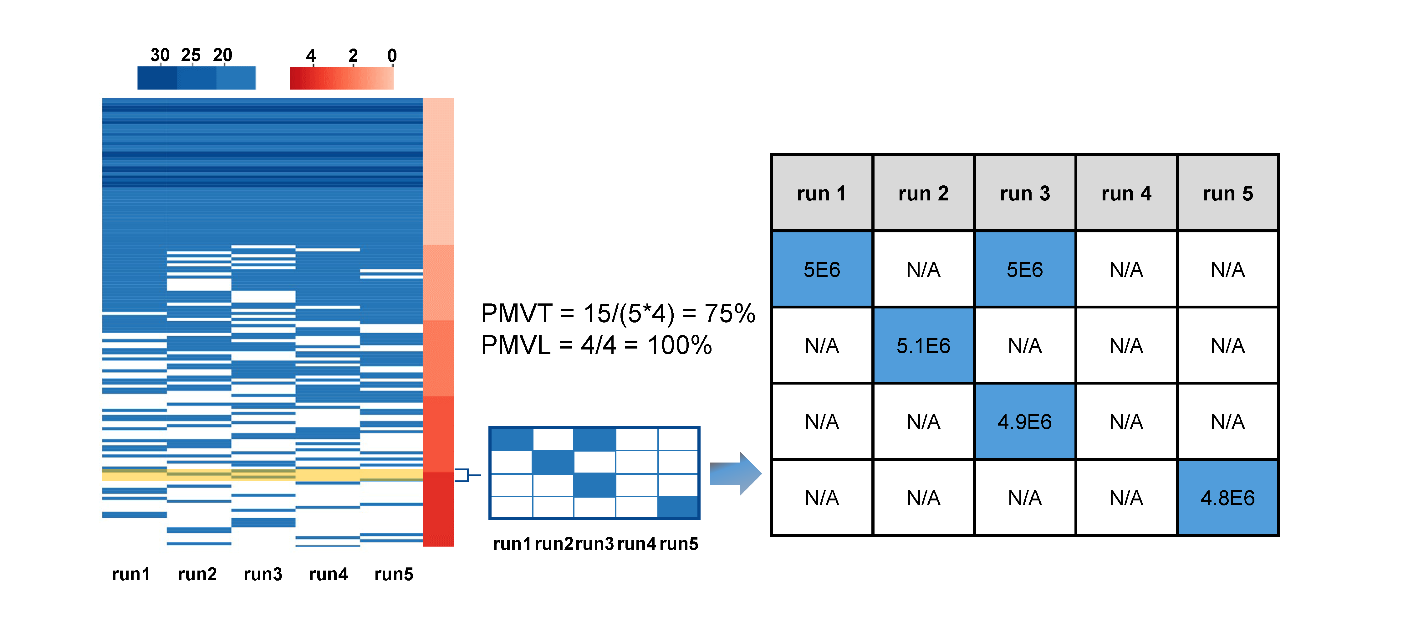
In this figure, a block indicates a quantitation value. Different shade of blue indicates different intensity values. The white color indicates missing value. We use red block to indicate the number of missing values in line. The deeper the red color, the more missing values in line.

# Supplementary Figure 7 An example of missing values from label-free data.


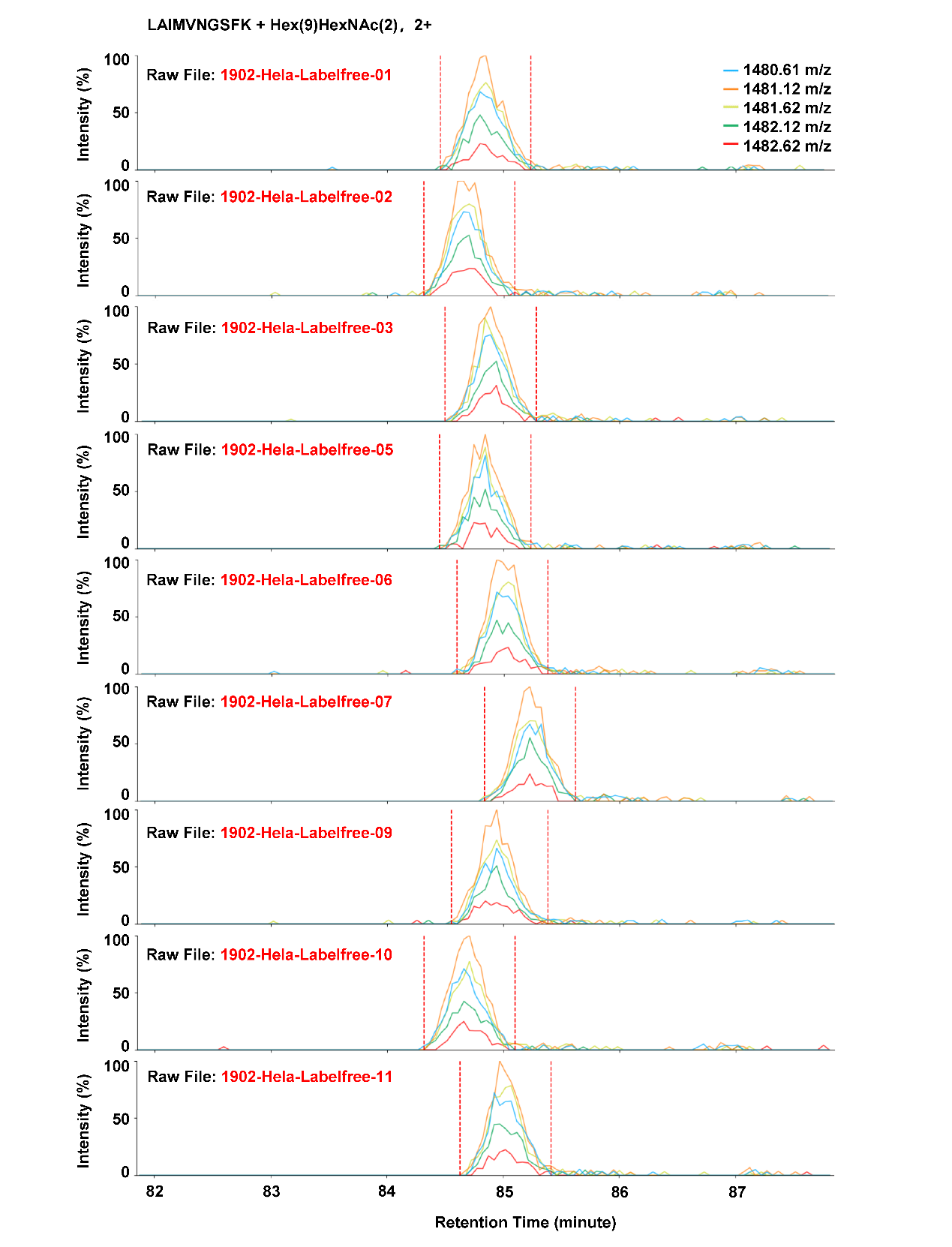
This figure illustrates the true signals of glycopeptide LAIMVNGSFK [Hex(9)HexNAc(2), 2+]. These signals are existed and could be extracted by pGlycoQuant, but missed by other software tools.

Supplementary Figure 8 Comparison of the quantitative performance of all software tools on SILAC-labeled 293T cell data at glycopeptide level in the aspect of Pearson correlation and standard deviation. (a) The correlation coefficient of quantitative intensity of each software. The lower left part is the scatter distribution diagram of intensity of light glycopeptide and heavy glycopeptide, and the upper right part is the calculated Pearson linear correlation coefficient. (b) The standard deviation of intensity ratio of each software. The lower left part is the violin diagram of intensity ratio of light glycopeptide and heavy glycopeptide, and the upper right part is the calculated standard deviation.


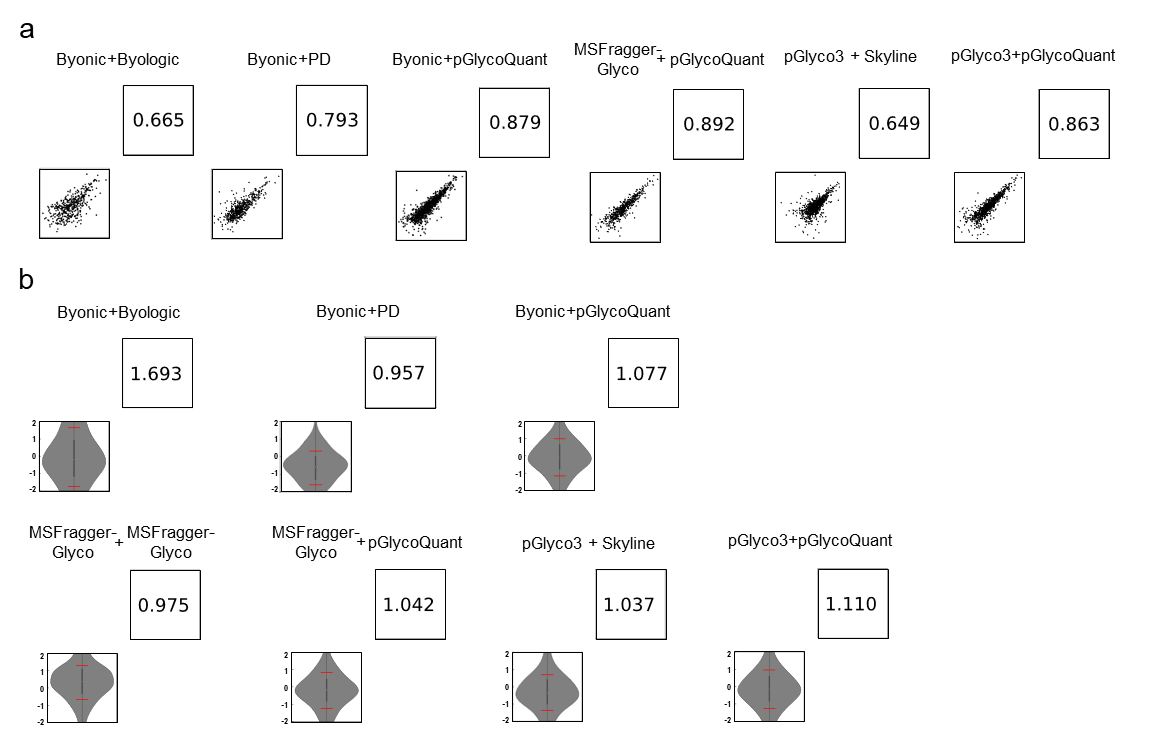
We evaluated the performance of quantitative results of each software on SILAC-labeled 293T cell data and proved that pGlycoQuant can achieve better quantitative accuracy. From the following figure a, it can be seen that the quantitative results reported by pGlycoQuant have a higher correlation. It should be noted that MSFragger-Glyco's quantitative results from running SILAC data are the intensity ratios of light and heavy glycopeptides, not the intensity values, so there is no MSFragger-Glyco correlation result. As shown in the following figure b, the quantitative result reported by pGlycoQuant has a lower standard deviation.

Supplementary Figure 9 Comparison of the quantitative performance of all software tools on label-free HeLa cell data at glycopeptide level in the aspect of Pearson correlation and standard deviation. (a) The correlation coefficient of quantitative intensity of each software. The lower left part is the scatter distribution diagram of intensity between two repeated data, and the upper right part is the calculated Pearson linear correlation coefficient. (b) The standard deviation of intensity ratio of each software. The lower left part is the violin diagram of intensity ratio between two repeated data, and the upper right part is the calculated standard deviation.

We evaluated the performance of quantitative results of each software on label-free data and proved that pGlycoQuant can achieve better quantitative accuracy. From the following figure a, it can be seen that the quantitative results reported by pGlycoQuant have a higher correlation. As shown in the following figure b, the quantitative result reported by pGlycoQuant has a lower standard deviation.


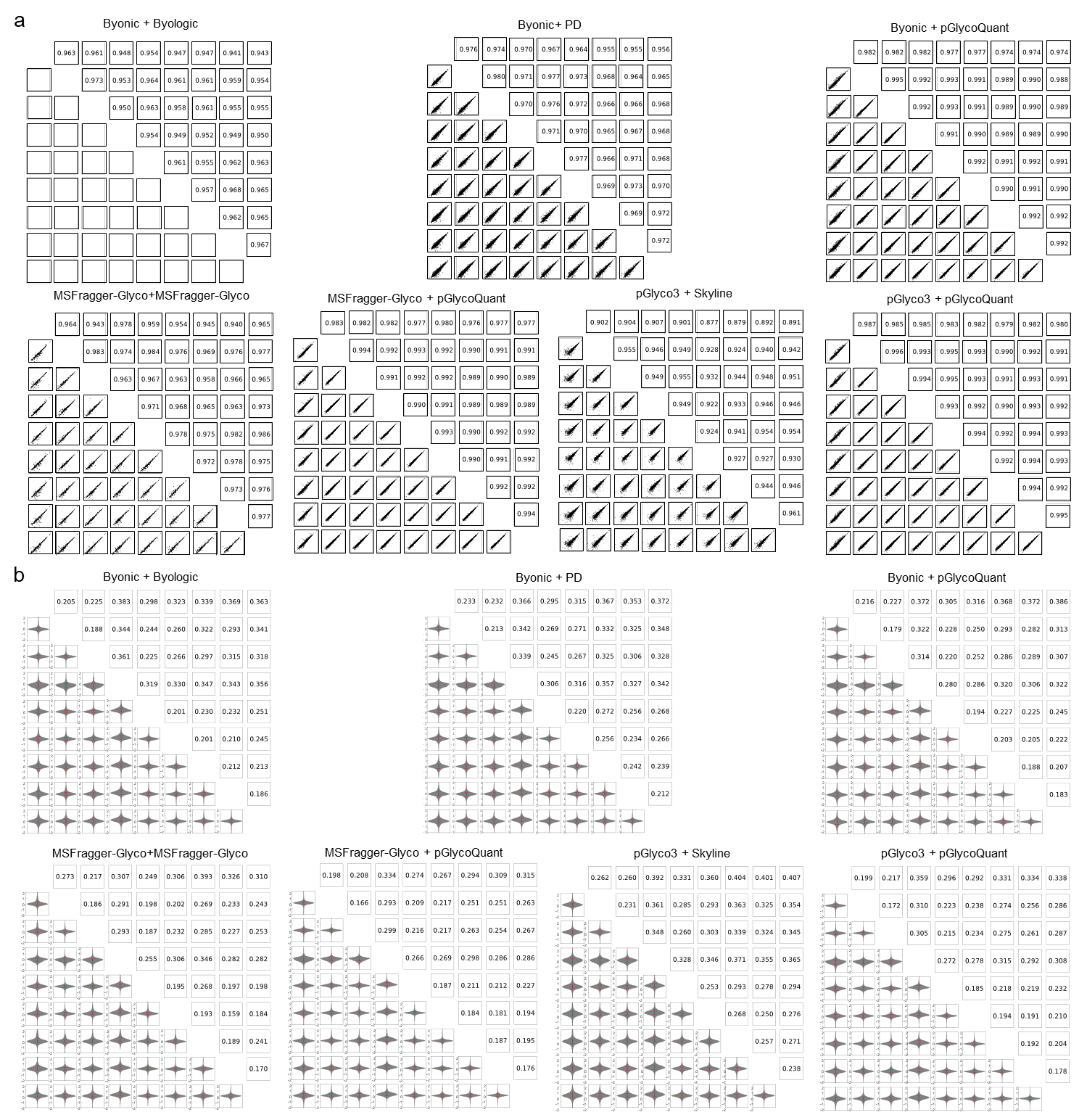


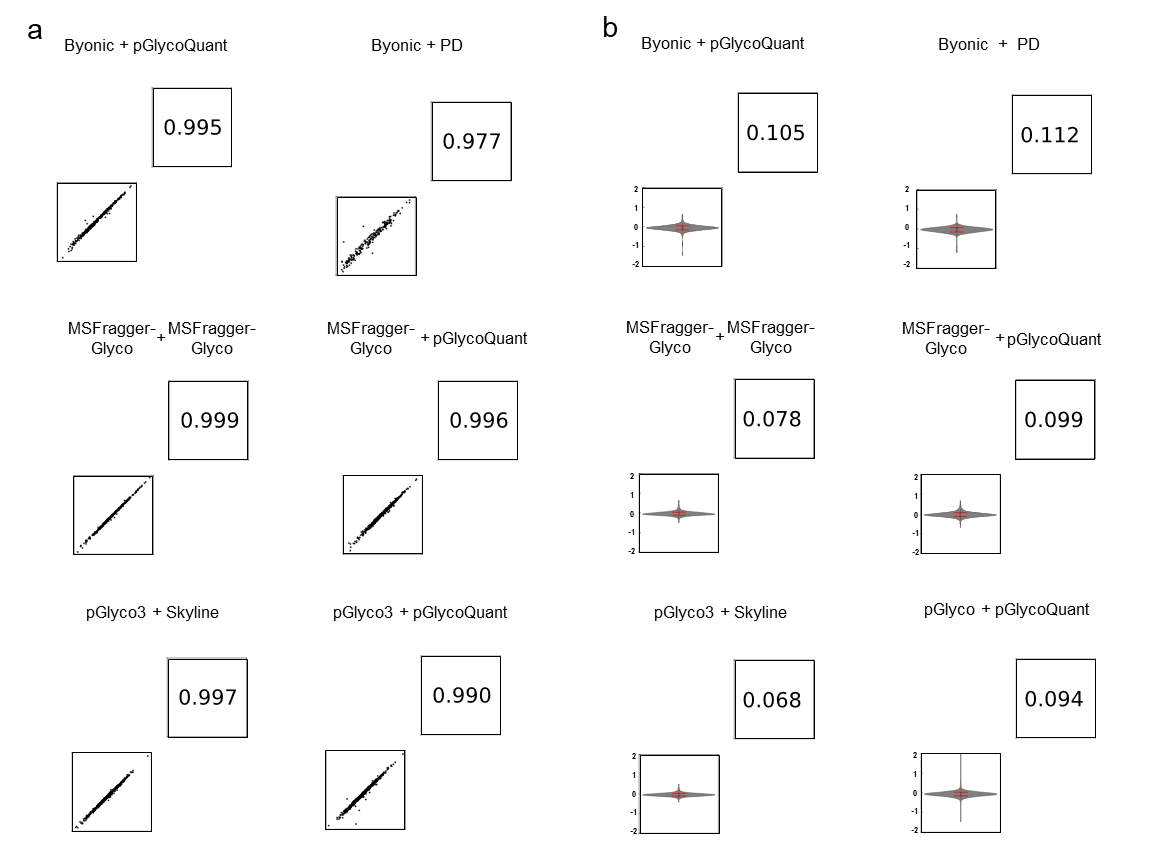
Supplementary Figure 10 Comparison of the quantitative performance of all software tools on TMT-labeled 293T cell data at glycopeptide level in the aspect of Pearson correlation and standard deviation. (a) The correlation coefficient of quantitative intensity of each software. The lower left part is the scatter distribution diagram of intensity of two labels, and the upper right part is the calculated Pearson linear correlation coefficient. (b) The standard deviation of intensity ratio of each software. The lower left part is the violin diagram of intensity ratio of two labels, and the upper right part is the calculated standard deviation.

Supplementary Figure 11 The quantitation results of the fold change-(de)glycoproteome data from IgG reported by different software tools. Histogram with fitting for visualization of the 5-fold change of the glycopeptide quantification results reported by different software portfolio (a-g), and visualization of quantitative ratio between deglycopeptide condition and glycopeptide condition reported by different software portfolio (h-n). The fold change-(de)glycoproteome data as that described in Supplementary Figure 5 and Supplementary Note 2 were used here to compare the different software tools. The results of pGlyco3+pGlycQuant portfolio, which have already been presented in Supplementary Figure 5, were also shown here for ease of comparison between the other methods.

**
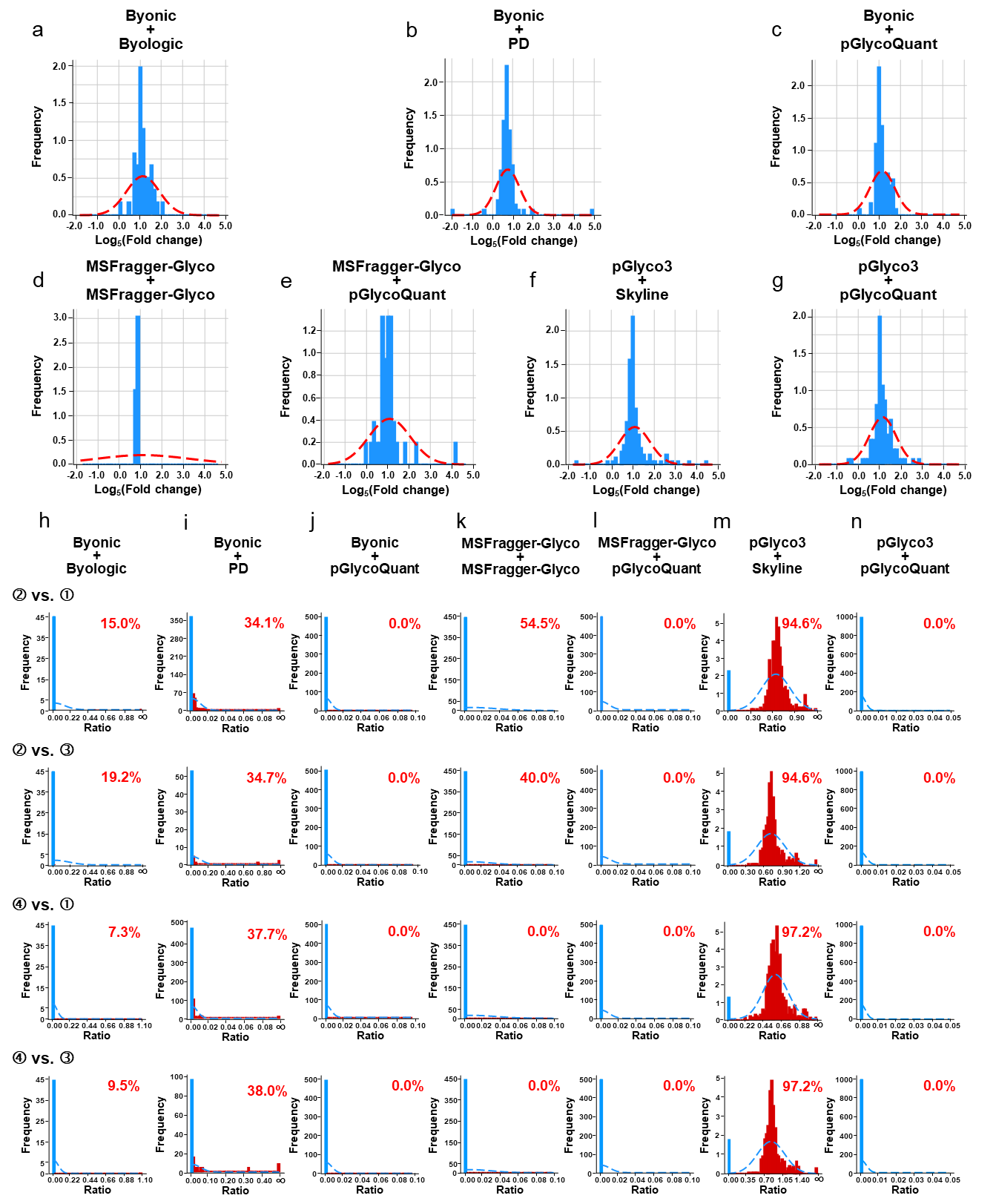
**

Supplementary Figure 12 The quantitation results of the fold change-(de)glycoproteome data from fission yeast reported by different software tools. Histogram with fitting for visualization of the 5-fold change of the glycopeptide quantification results reported by different software portfolio (a-g), and visualization of quantitative ratio between deglycopeptide condition and glycopeptide condition reported by different software portfolio (h-n). The fold change-(de)glycoproteome data as that described in Supplementary Figure 5 and Supplementary Note 2 were used here to compare the different software tools. The results of pGlyco3+pGlycQuant portfolio, which have already been presented in Supplementary Figure 5, were also shown here for ease of comparison between the other methods.

**
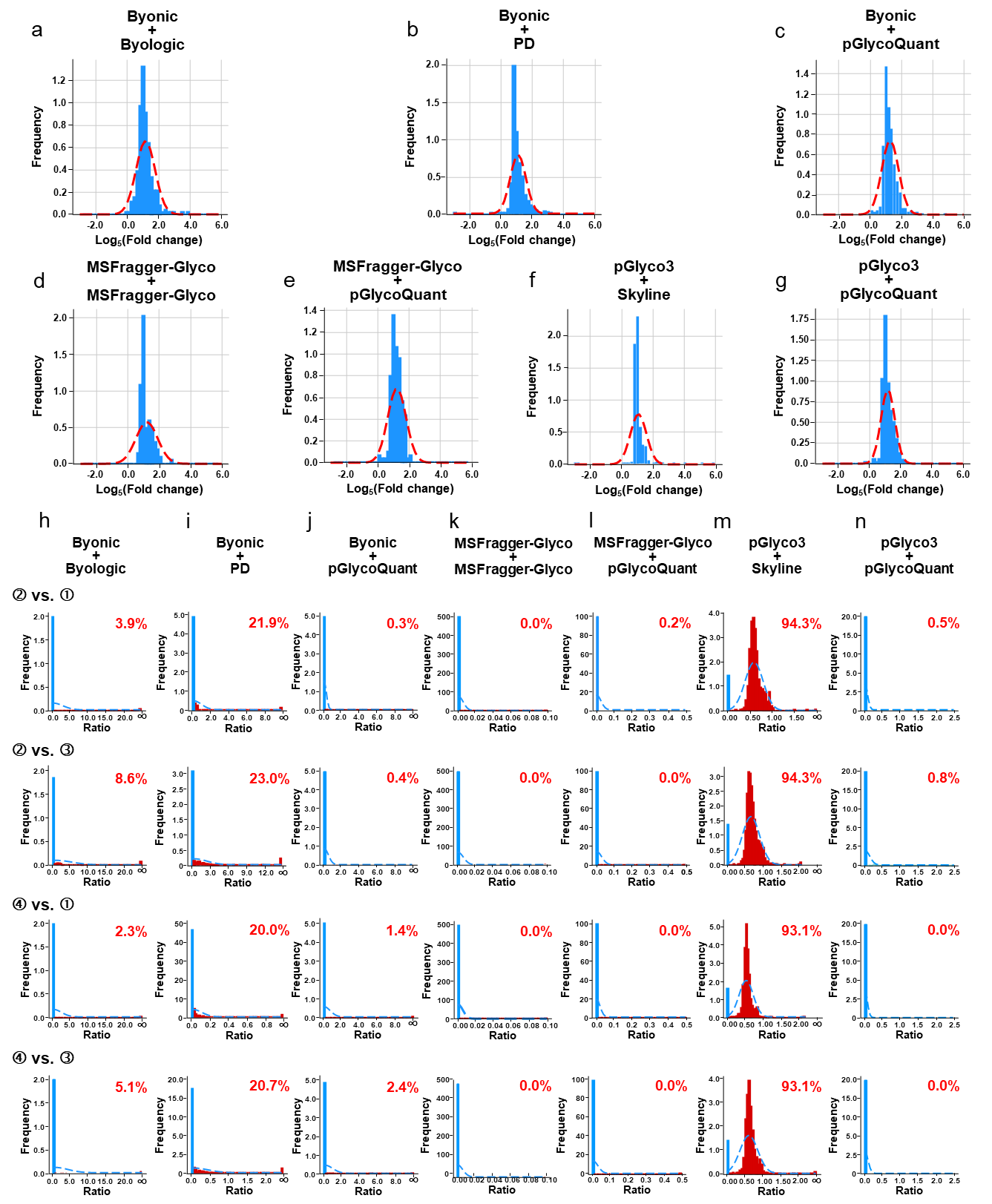
**

Supplementary Figure 13 The quantitation results of the fold change-(de)glycoproteome data from human serum reported by different software tools. Histogram with fitting for visualization of the 5-fold change of the glycopeptide quantification results reported by different software portfolio (a-g), and visualization of quantitative ratio between deglycopeptide condition and glycopeptide condition reported by different software portfolio (h-n). The fold change-(de)glycoproteome data as that described in Supplementary Figure 5 and Supplementary Note 2 were used here to compare the different software tools. The results of pGlyco3+pGlycQuant portfolio, which have already been presented in Supplementary Figure 5, were also shown here for ease of comparison between the other methods.

**
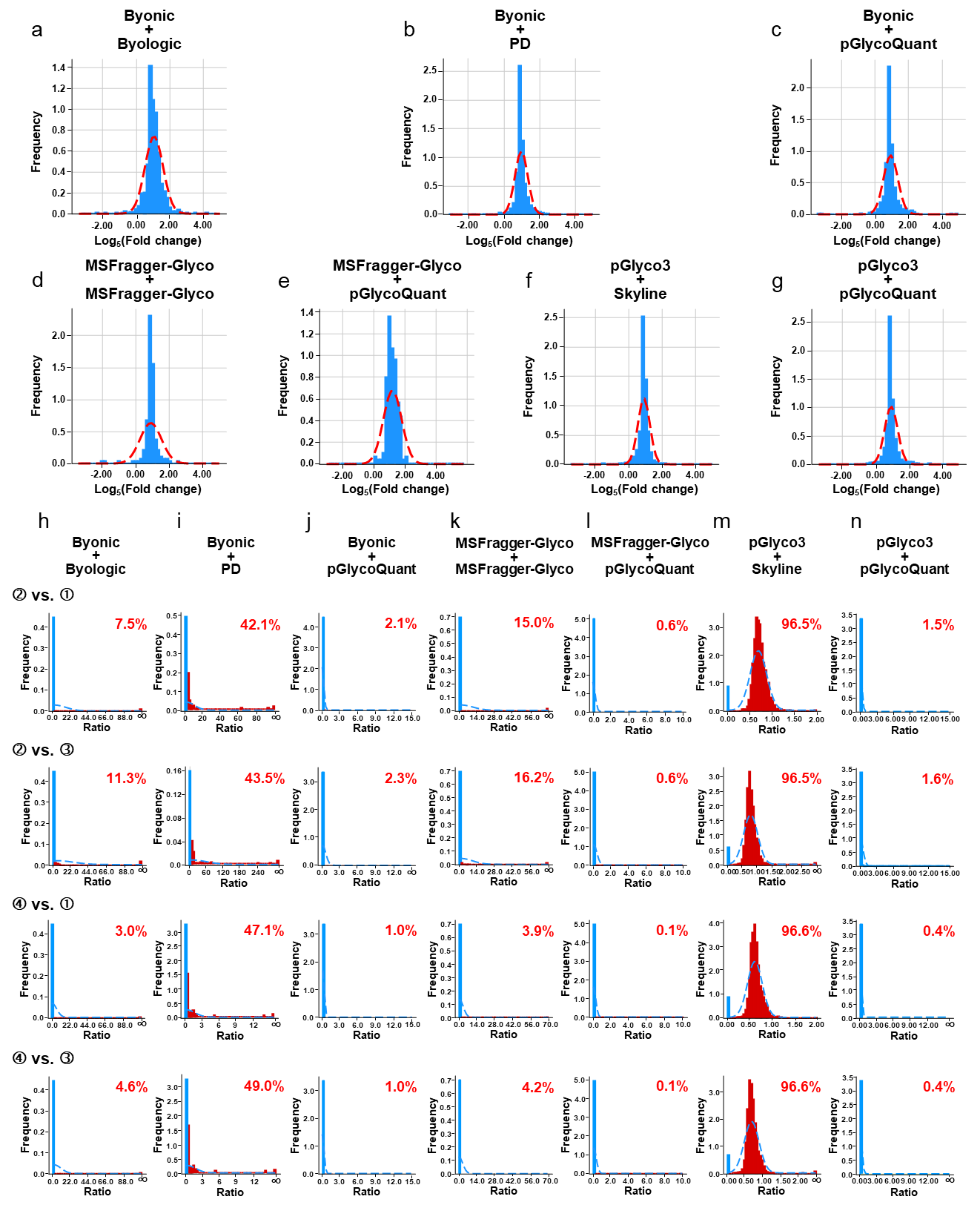
**

**
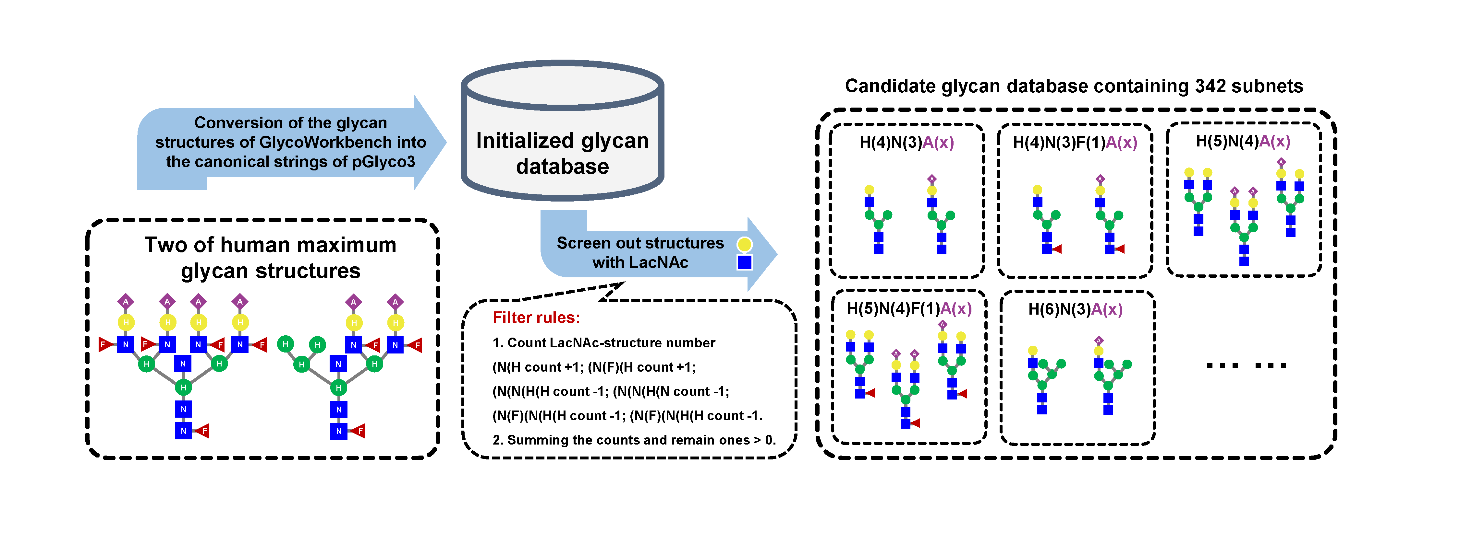
**Supplementary Figure 14 The candidate glycan database constructed for MIR analysis. A candidate glycan database was constructed from the two of maximum human glycan structures through initialized glycan database and several filter rules, and then grouped into several subnets. Each subnet contains glycans with the same glycan infrastructure. The term of “glycan infrastructure” used here refers to a glycan structure excluding sialic acid units.

Supplementary Figure 15 The experimental design for validation of MIR using benchmarked N-glycopeptides. (a) A list of ten synthetic N-glycopeptides as benchmarked N-glycopeptide for validation. (b) The experimental pipeline.


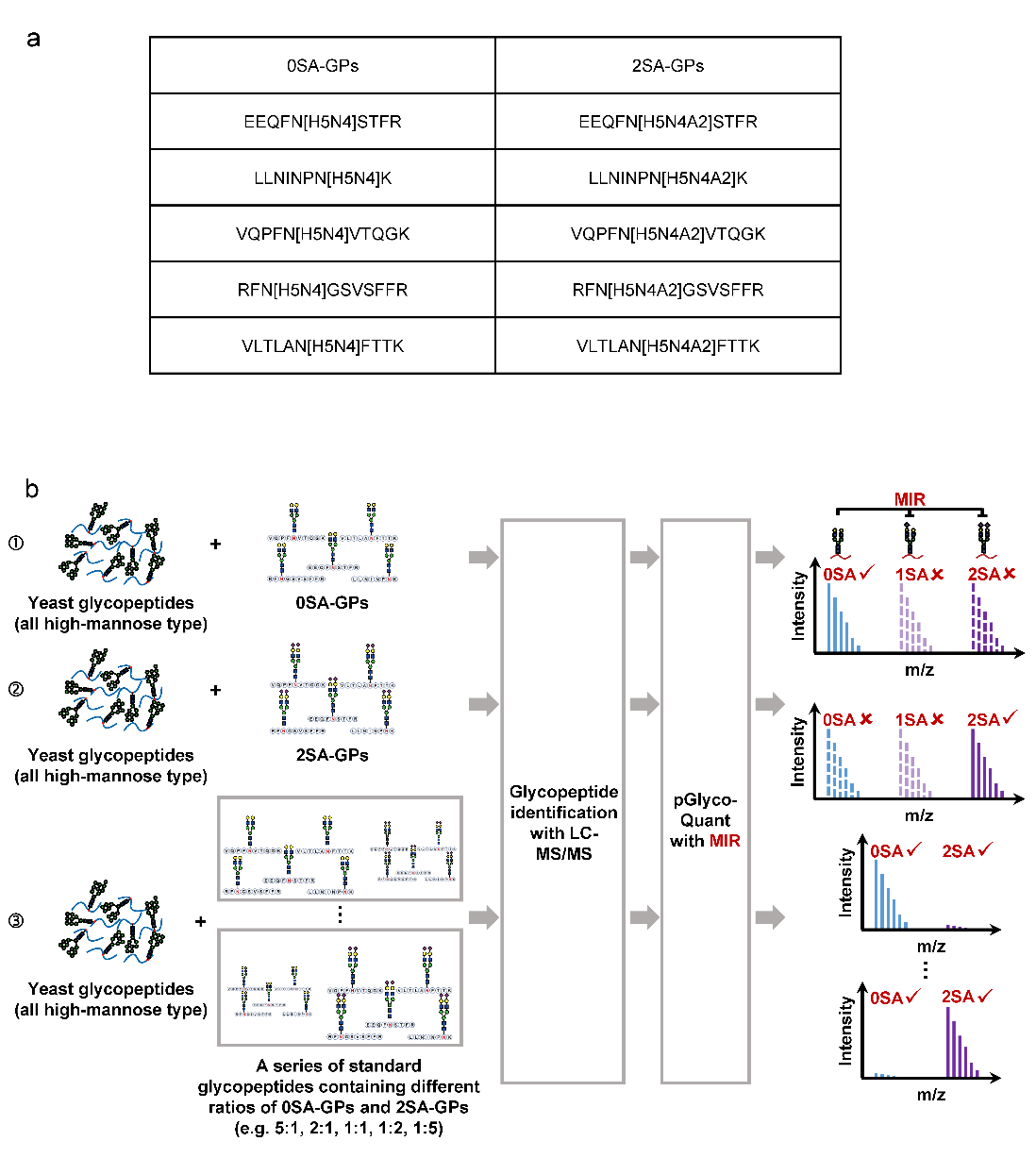
We first synthesized five N-glycopeptides attached with glycan H(5)N(4) (0SA-GPs) and five N-glycopeptides attached with glycan H(5)N(4)A(2) (2SA-GPs). Then we mixed the synthetic glycopeptides with yeast glycopeptides (all high-mannose types) to mimic complex samples, and performed MIR analysis on each of the following mixtures: 1) mixture 1 containing 0SA-GPs and yeast glycopeptides, 2) mixture 2 containing 2SA-GPs and yeast glycopeptides, 3) a series of mixtures containing 0SA-GPs and 2SA-GPs with different ratios (0SA-GPs:2SA-GPS, 1:5, 1:2, 1:1, 2:1, 5:1) and yeast glycopeptides.

#
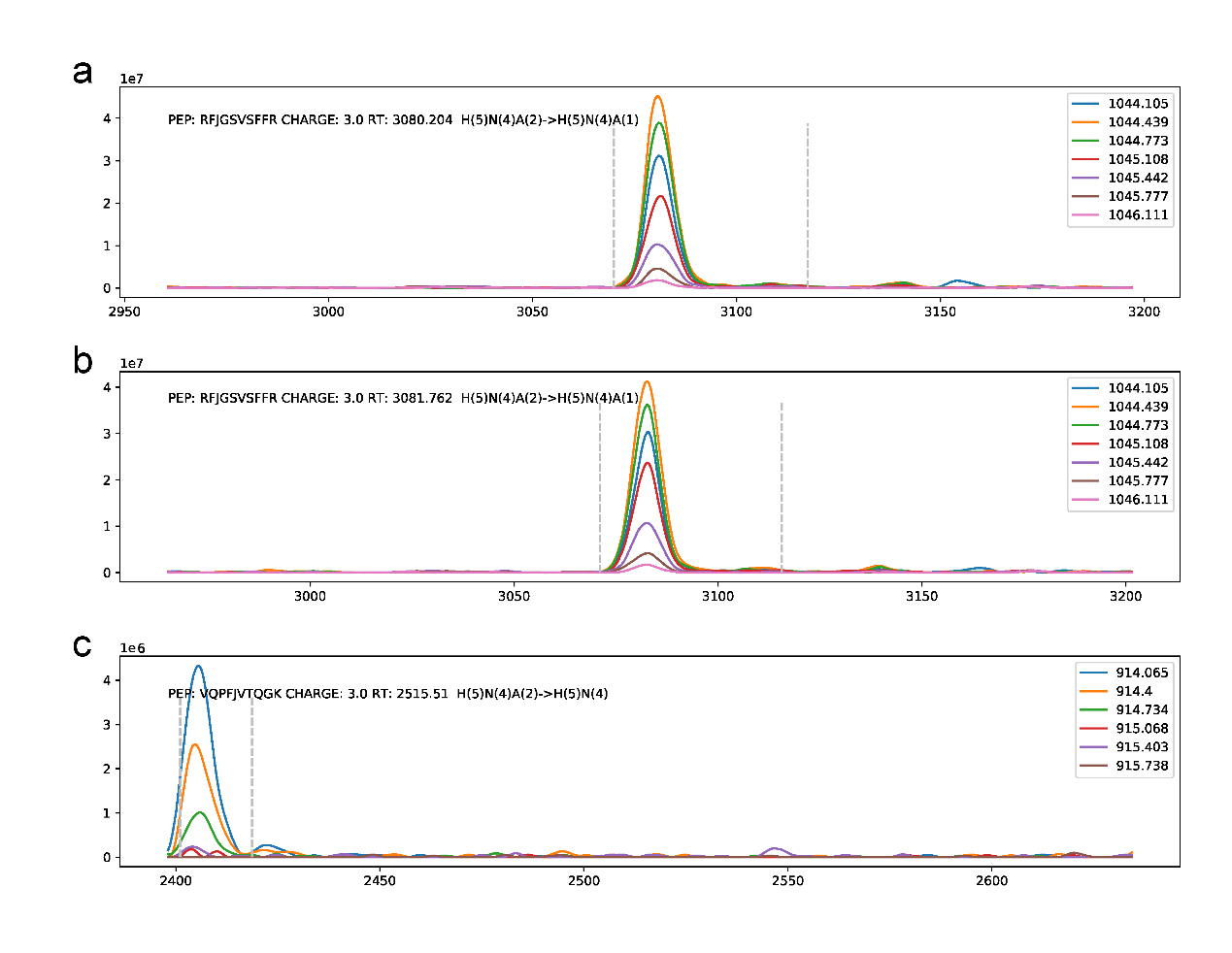
Supplementary Figure 16 The isotope distribution of the glycopeptide evidences in MS1 scans for the three abnormal scores for 1SA-GPs in Figure 3f.

Supplementary Figure 17 MIR analysis of N-glycopeptides in human IgG. (a) Glycopeptide evidences with MIR matching score over 5 were screened out. The center line indicates the median. The boxes indicate the interquartile ranges (IQRs), and the whiskers indicate 1.5 × IQR values. All outliers are shown. The gray dotted lines indicate MIR matching score of 5. (b)The number of quantified glycopeptides of human IgG by pGlycoQuant with MIR analysis.


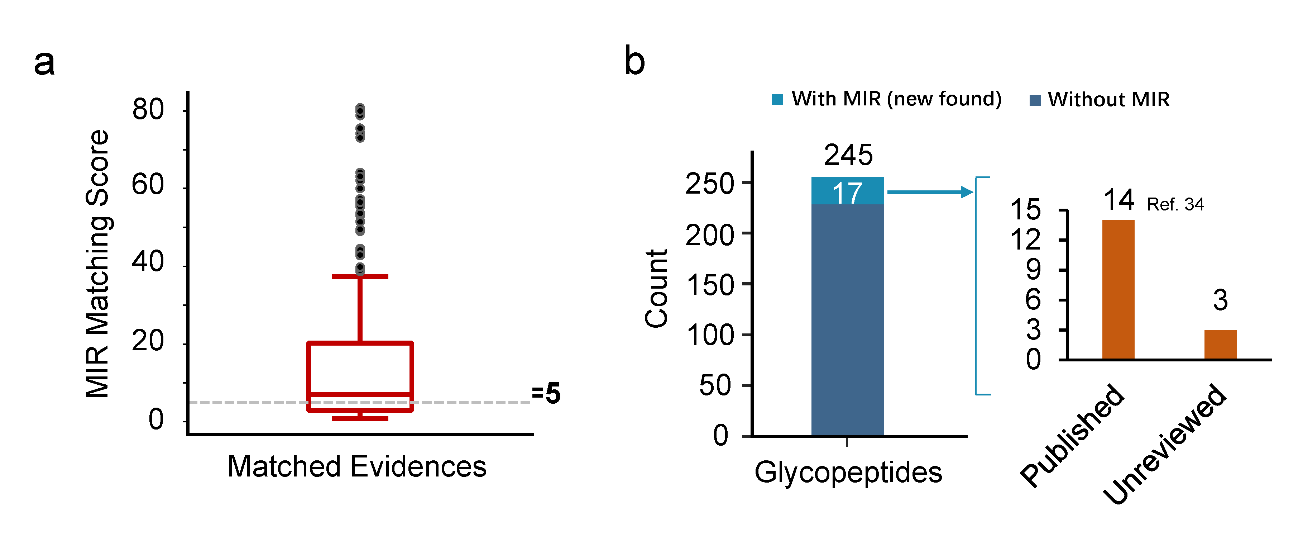


**
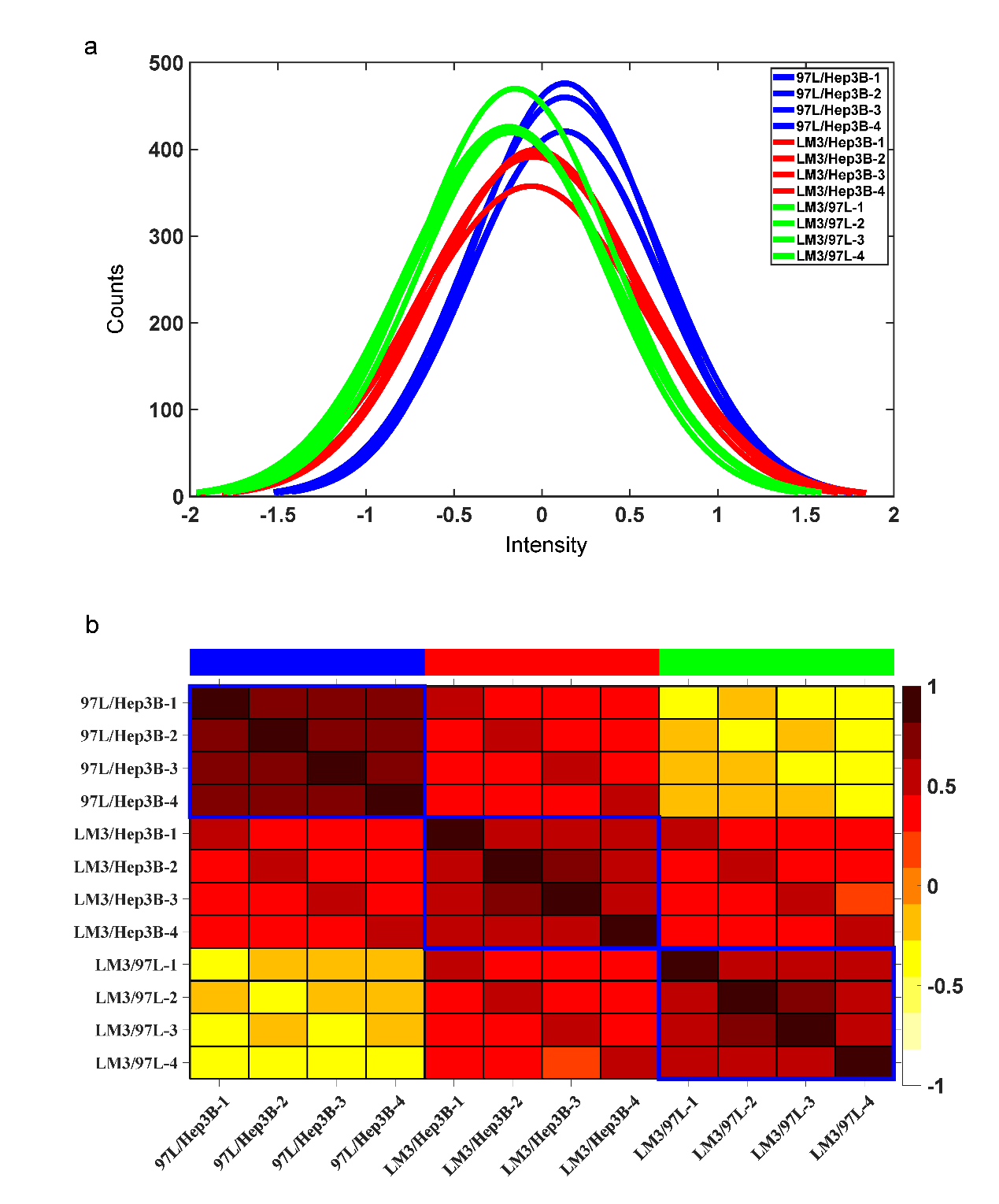
**Supplementary Figure 18 High repeatability of the proteome quantitative results among three cell lines. (a) The normal distribution fitting line. (b) A correlation heat map of proteome quantitative ratio between different replicates.

**
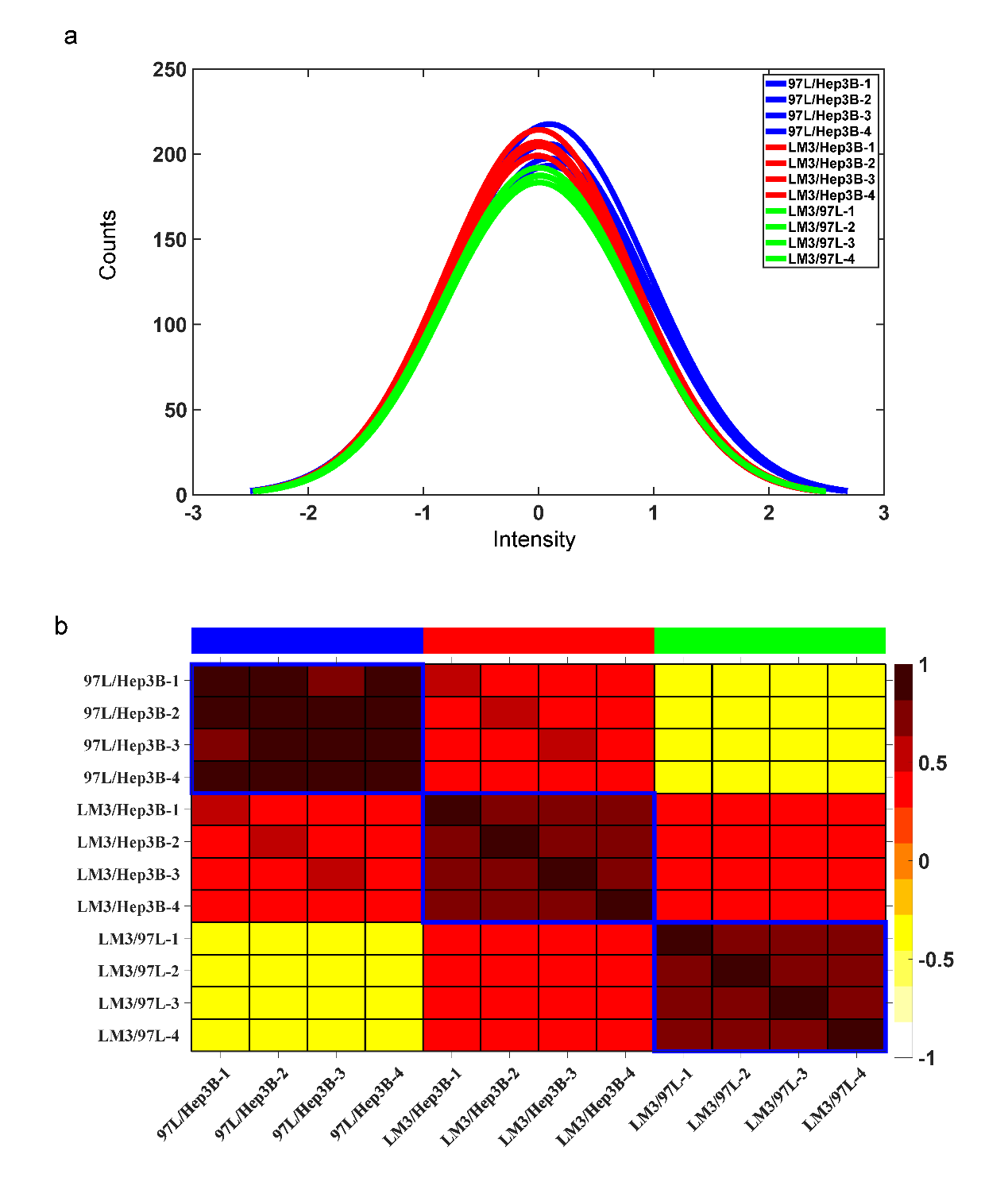
**Supplementary Figure 19 High repeatability of the intact glycopeptide quantitative results among three cell lines. (a) The normal distribution fitting line. (b) A correlation heat map of intact glycopeptide quantitative ratio between different replicates.

Supplementary Figure 20 Gene ontology (GO) analyses of proteome on cellular component (a), molecular function (b), and biological process (c). Gene set enrichment p-values were calculated using Fisher’s exact test and adjusted Benjamini-Hochberg (BH) FDR-controlling method for multiple testing (Adjust P.value <0.05).

**
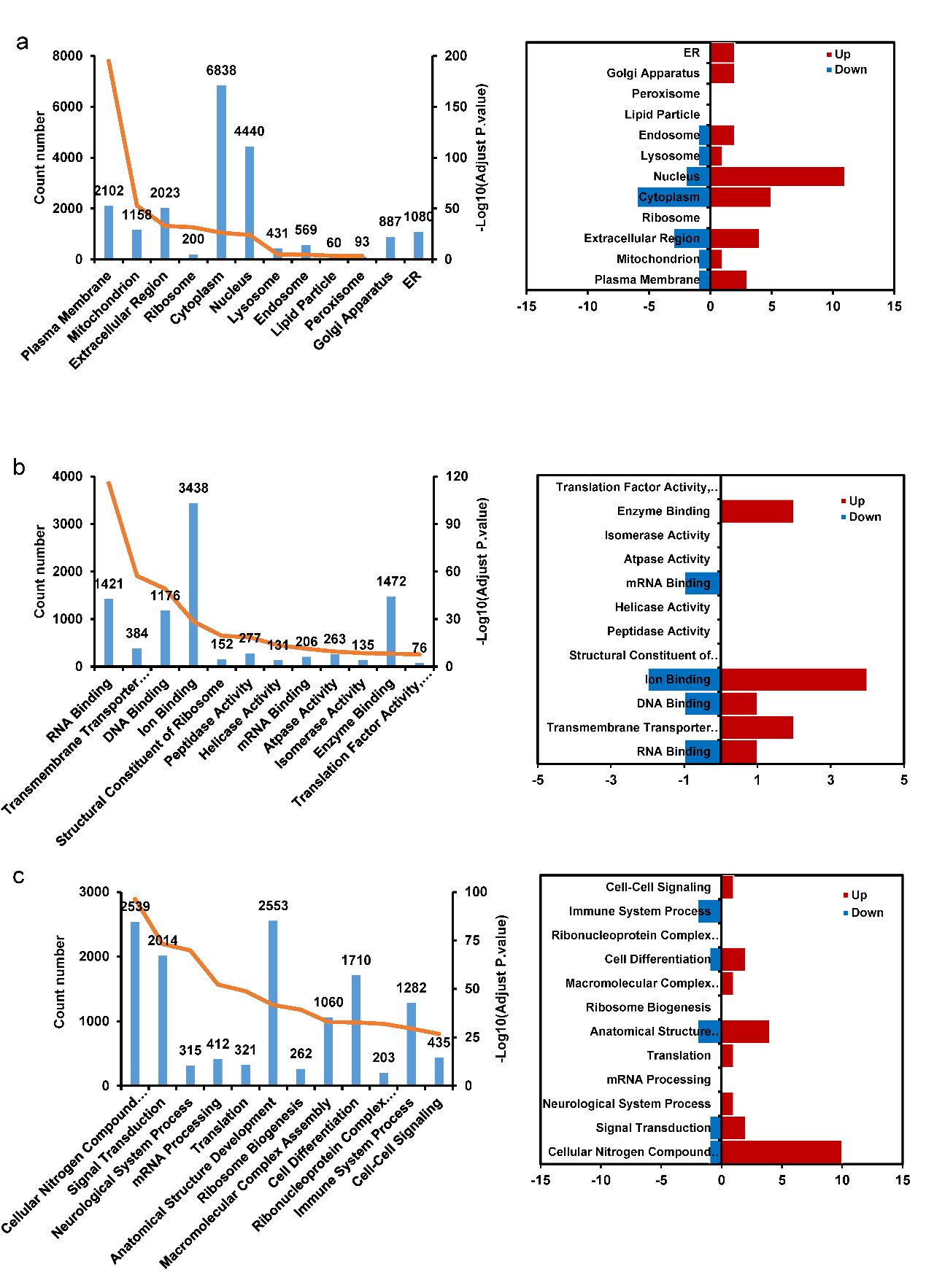
**

**
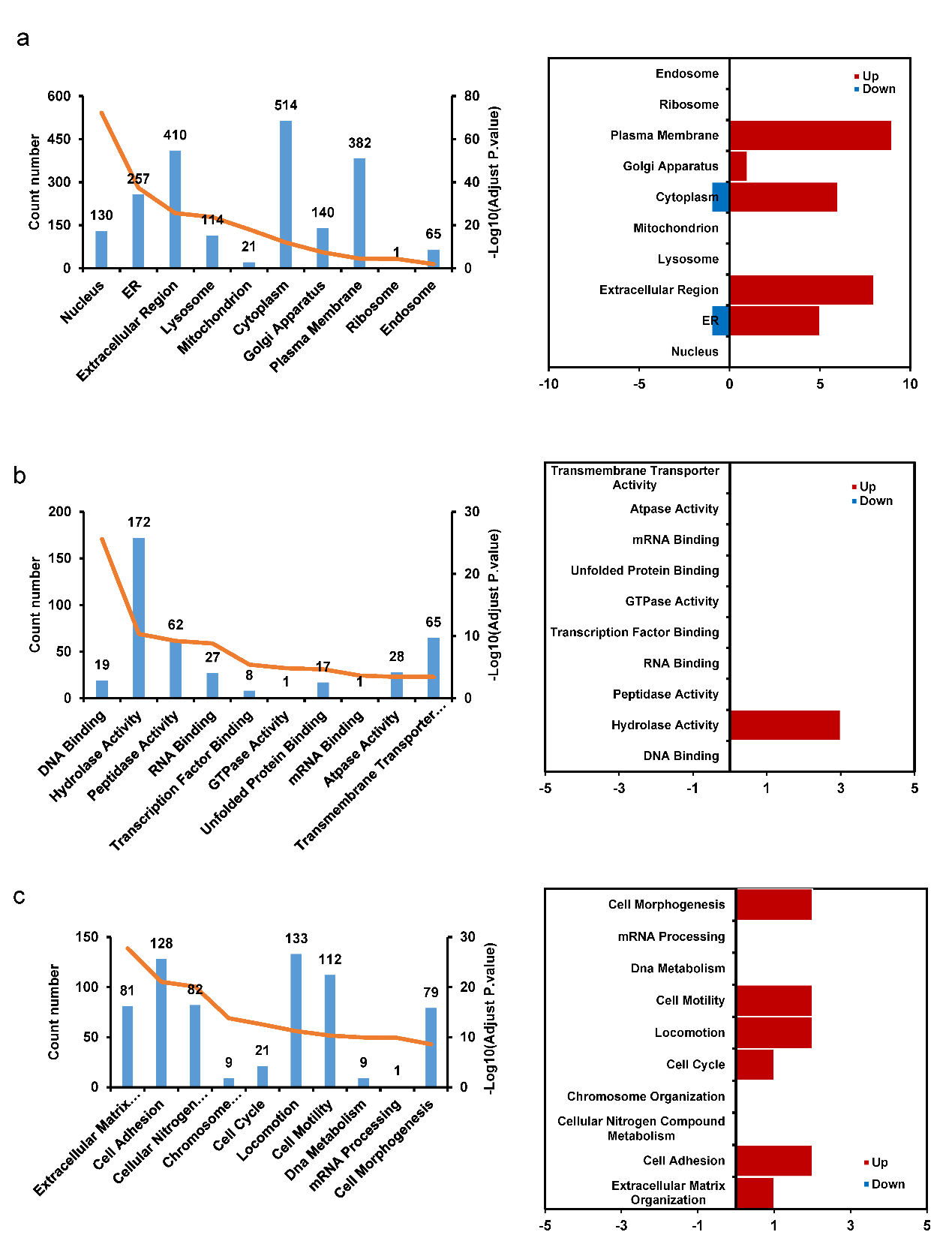
**Supplementary Figure 21 Gene ontology (GO) analyses of glycoproteome on cellular component (a), molecular function (b), and biological process (c). Gene set enrichment p-values were calculated using Fisher’s exact test and adjusted Benjamini-Hochberg (BH) FDR-controlling method for multiple testing (Adjust P.value <0.05).

**
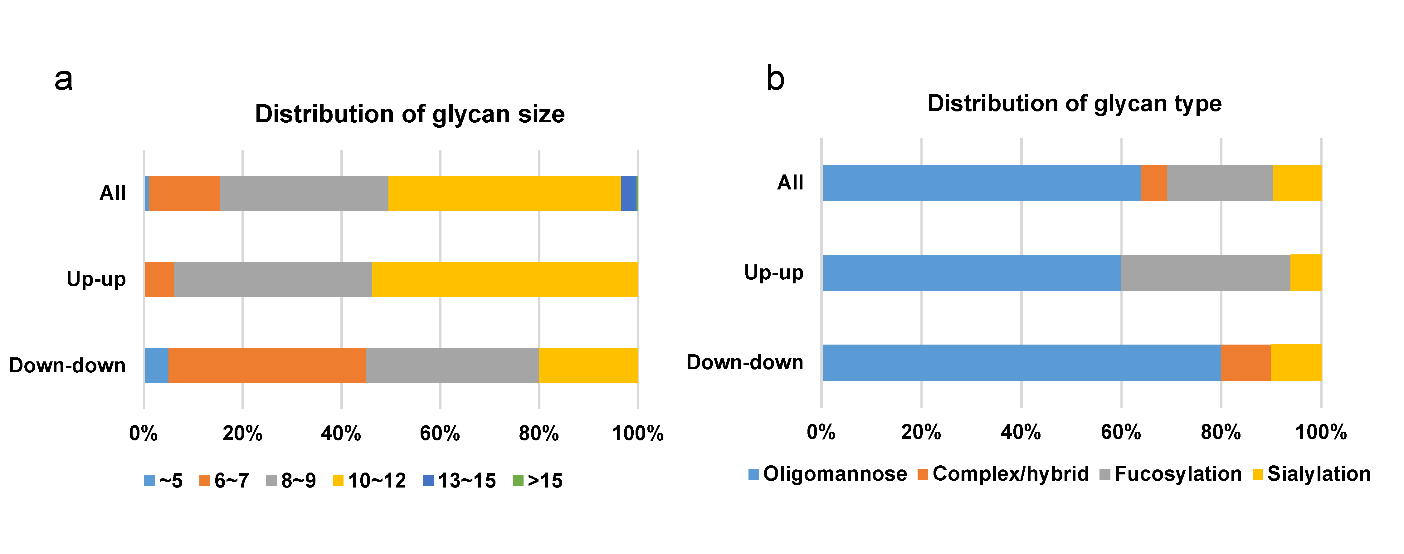
**Supplementary Figure 22 Distribution of glycan size and glycan type in three HCC cell lines. (a) Distribution of glycan size (the number of saccharides units on a glycan) in different categories. (b) Distribution of different glycan types, including oligomannose, complex/hybrid, fucosylation, and sialylation in categories. All is for all intact glycopeptides quantified in HCC cell lines. Up-up is for uniformly upregulated intact glycopeptides in three cell lines with increased metastatic potential. Down-down is for uniformly downregulated intact glycopeptides in three cell lines with increased metastatic potential. In the glycan classification, Complex/Hybrid is for the complex/hybrid-type glycans with no fucose and sialic acid. Oligomannose is for the glycans only containing mannose monosaccharide except for the core structure. Fucosylated glycans that contain sialic acid moieties are classified as Fucosylation instead of Sialylation.

**
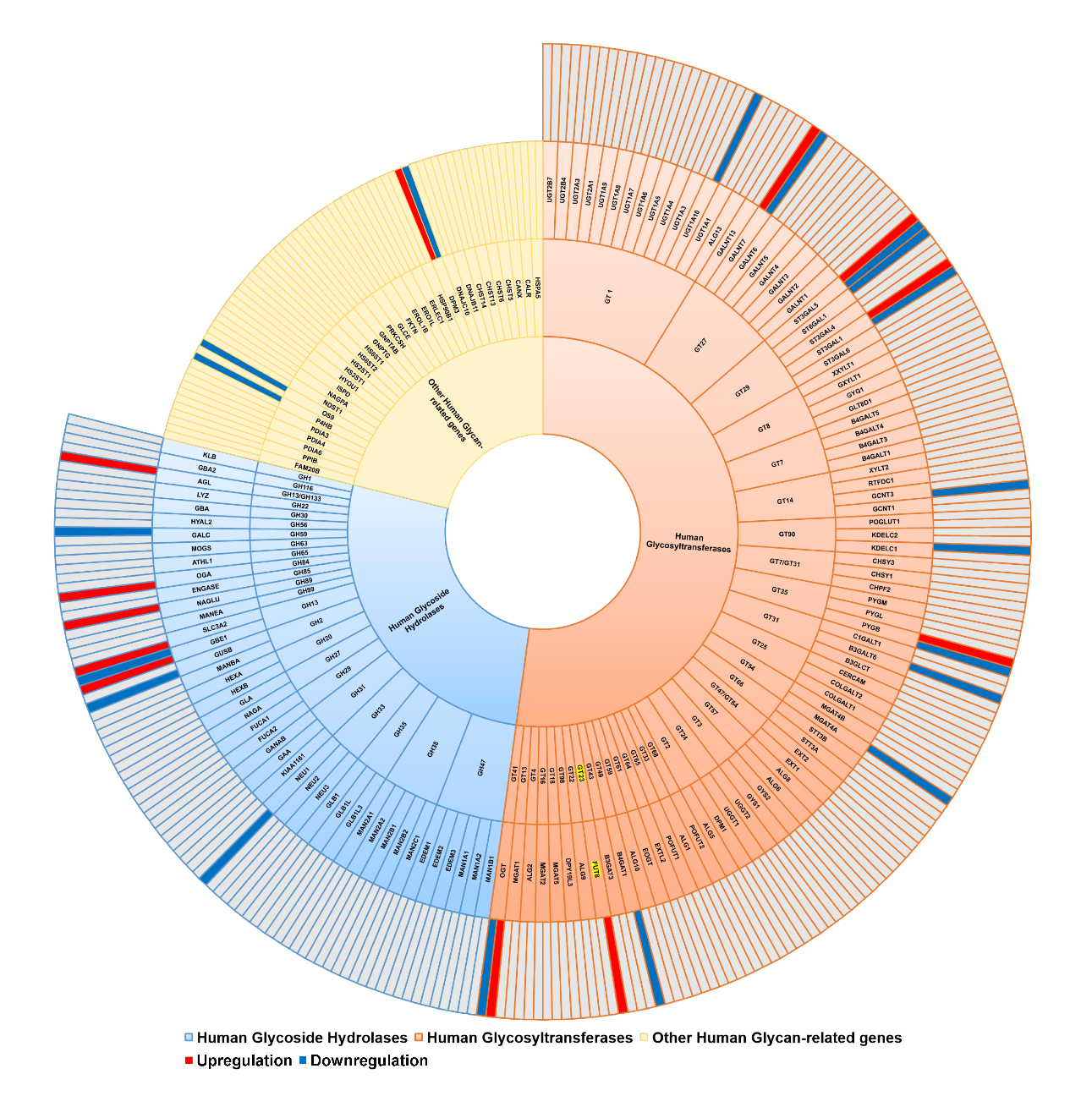
**Supplementary Figure 23 Differential expressed proteins of glycan-related genes in three HCC cell lines. Blue fan represents human glycoside hydrolases. Orange fan represents human glycosyltransferases. Yellow fan represents other human glycan-related genes. Each glycan-related gene has two bars in the outermost circle. In the clockwise direction, the first bar represents the change of the protein in 97L/Hep3B, and the second bar represents the change of the protein in LM3/97L. Red bar stands for the upregulation. Blue bar stands for the downregulation. FUT8, which is yellow-highlighted, is a core-fucosylation transferase and is selected as the target glycosyltransferase in this study. See the Supplementary Data 5 for the related data.

**
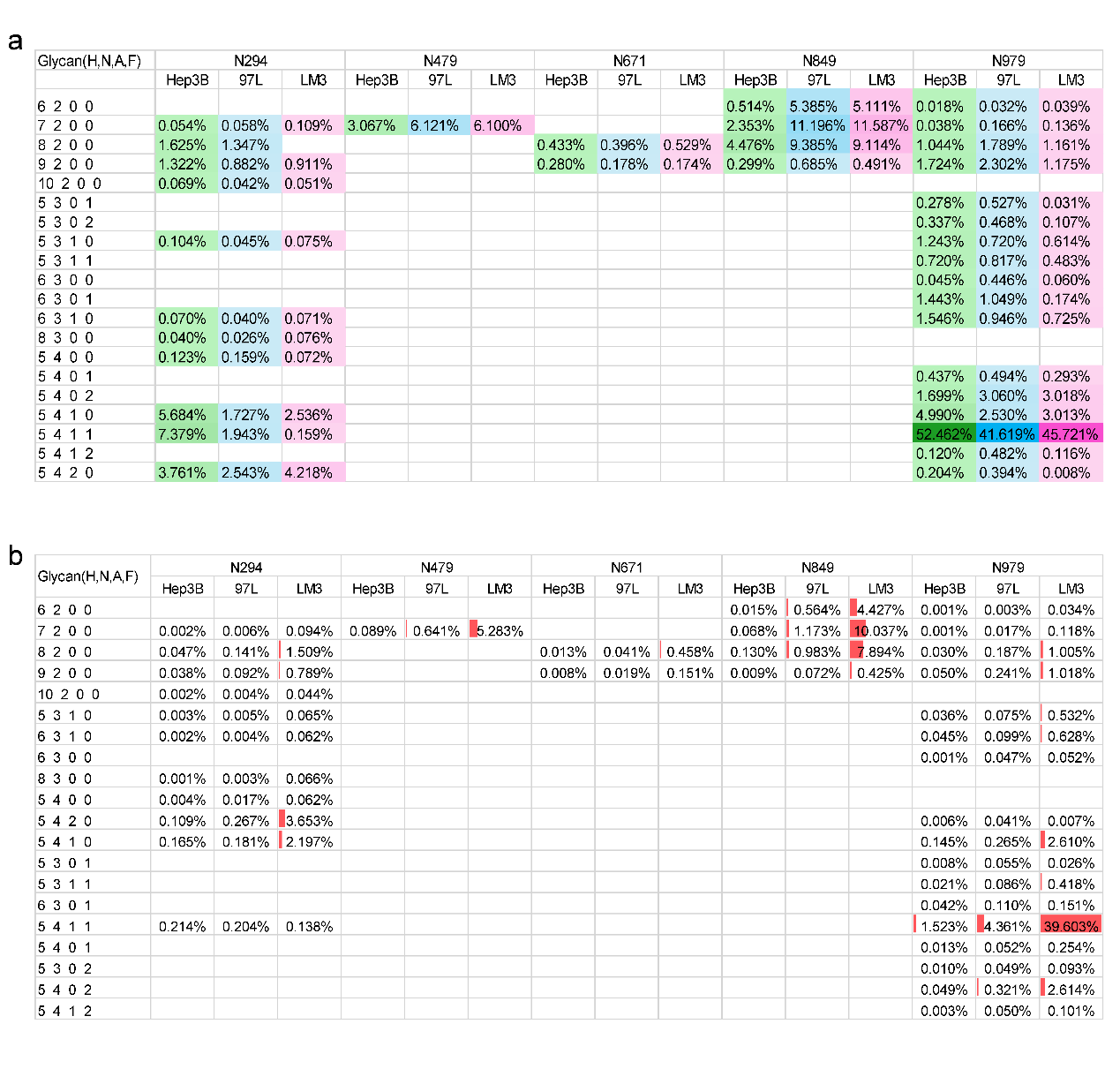
**Supplementary Figure 24 Site-specific glycosylation level of L1CAM in three HCC cell lines after normalization within a cell line or among three cell lines. (a) is a table of the percentage of a site-specific glycan intensity to the total intensity of site-specific glycans in a cell line. (b) is a table of the percentage of a site-specific glycan intensity to the total intensity of site-specific glycans in three cell lines.

Supplementary Figure 25 A comparison of differential intact glycopeptides without and with normalization to protein abundance. Volcano map of differential intact glycopeptides without (a) and with (b) normalization to protein abundance. Significant differential expression in the volcano plots were calculated by two-sided t-test and adjusted P-values were calculated by Benjamini-Hochberg (BH) FDR-controlling method for multiple testing. Box diagram with four replicates for the quantitation of intact glycopeptides without (c) and with (d) normalization to protein abundance. In the box plots of c and d, the center line indicates the median. The boxes indicate the interquartile ranges (IQRs), and the whiskers indicate 1.5 × IQR values. All outliers are shown. (e) The Changes of fucosylation at site 979 of L1CAM in different HCC cell lines. The abundance of fucosylation at L1CAM-site 979 after normalization to L1CAM protein abundance showed no significant changes in LM3/97L, demonstrating increased fucosylation abundance at site 979 was caused by the protein abundance changes from low metastatic potential to high metastatic potential. Significant changes were calculated by two-sided t-test and adjusted P-values were calculated by Benjamini-Hochberg (BH) FDR-controlling method for multiple testing. The detailed data are shown in the Supplementary Data 7.


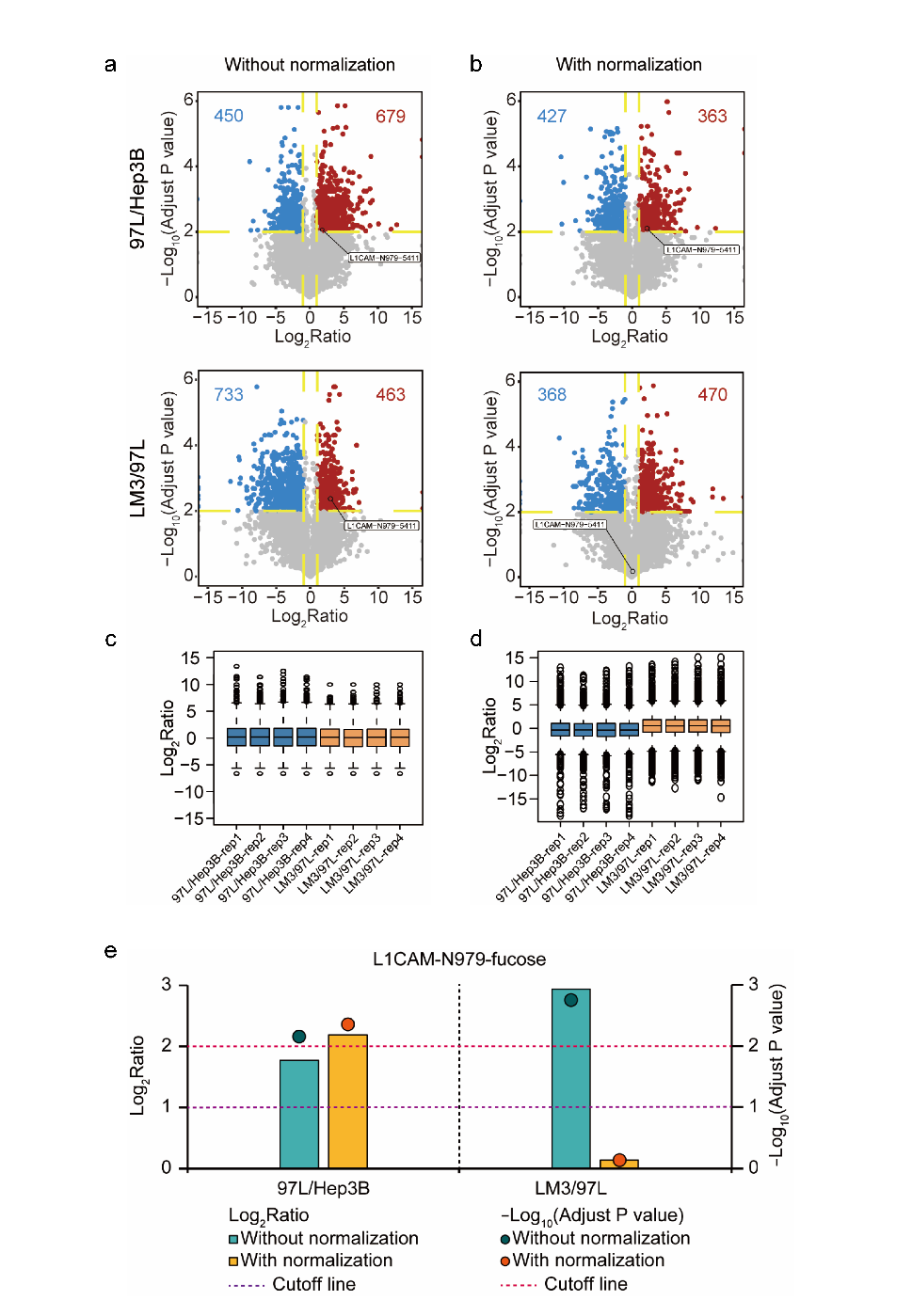


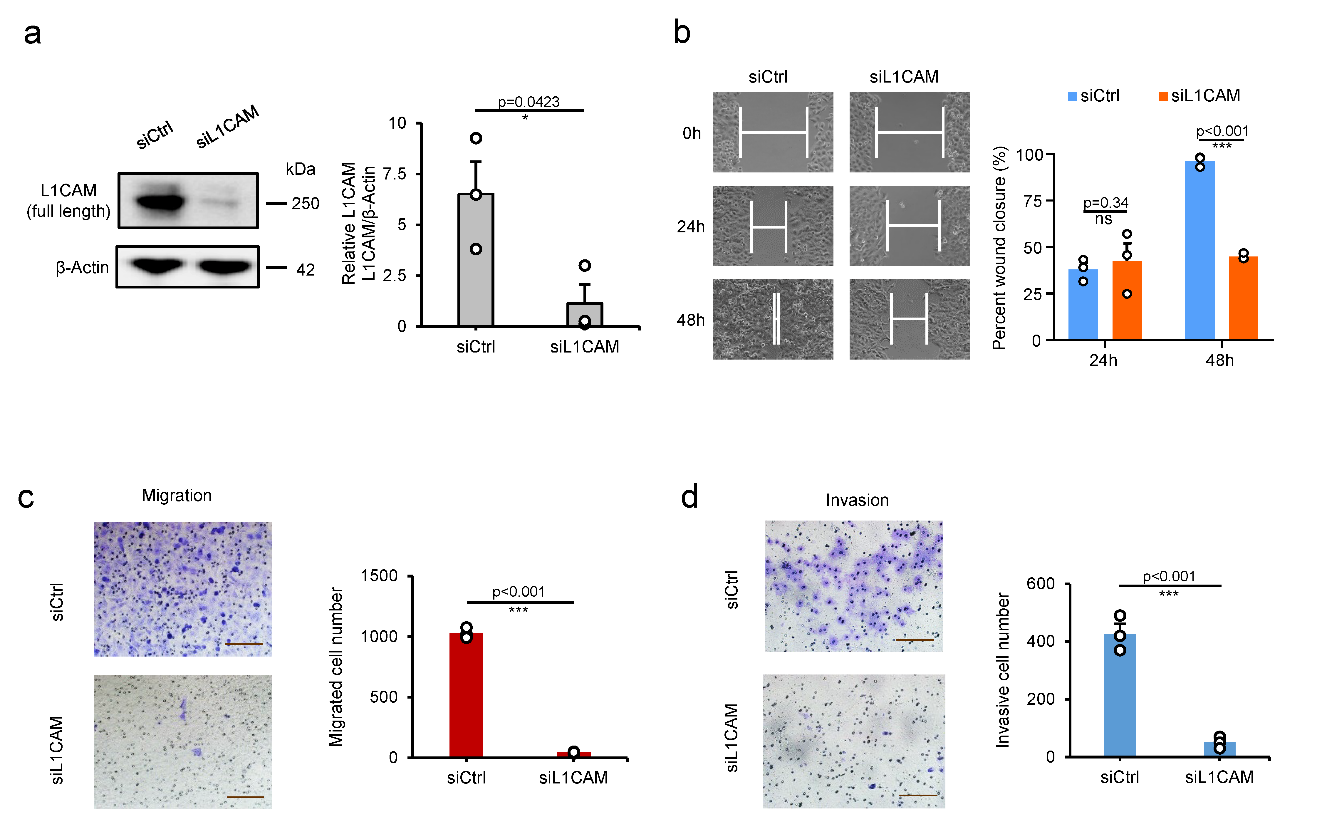
Supplementary Figure 26 Silencing L1CAM inhibited migration and invasion ability of LM3 cells. (a) Western blot of L1CAM levels in 97L cells transfected with negative control (Ctrl) and L1CAM siRNA. (b) Wound healing assay in monolayers of knocked down LM3 cells (siCtrl and siL1CAM). Scratch area of the cells were detected with an inverted microscope (10×). (c) Transwell migration assay (upper) and (d) matrigel invasion assay (down) (Scale bar = 100 µm) in knocked down LM3 cells (siCtrl and siL1CAM). The grayscale values of the western blot data (a) and scratch area of the wound healing assay were measured by Image J. β-Actin was used for the normalization of loading in all western blot data (a). The data (a-d) are presented as mean values ± SEM from three independent experiments. P values were determined by one-way ANOVA with Tukey’s test or two-tailed unpaired t-test. ns not significant, ∗p < 0.05, ∗∗p < 0.01, and ∗∗∗p < 0.001 compared to the control (n = 3). See the Source Data file for the exact P values. Source data are provided as a Source Data file.

# Supplementary Note 1 Experimental methods for the two-glycoproteome data obtaining.

As the experimental design shown in the Supplementary Figure 4, we enriched glycopeptides from fission yeast and human serum samples and analyzed by LC-MS/MS, respectively, and then performed MBR analysis between the two samples.

**Human sample preparation**

Informed consent was obtained under protocols that were approved by an institutional review board. The research followed the tenet of the Declaration of Helsinki and was approved by the Ethics Committee of the Fudan University. A total of ten healthy human blood samples were collected, immediately placed on ice for 30 minutes and then centrifuged at 2,000 g for 15 minutes. The supernatant was collected. A pooled serum specimen mixed from equal volume of the supernatant was used in this study. A volume of 15 μL of the pooled serum was added to 185 μL of 50 mM NH_4_HCO_3_, and placed in boiling water and ice 40 times alternately to denature the proteins. The proteins were then reduced with 10 mM DTT at 37 °C for 1 h and alkylated with 20 mM IAA for 0.5 h at room temperature in the dark. Then the proteins were digested into peptides by incubating with trypsin (a final enzyme-to-substrate ratio of 1:50) at 37 °C overnight, and then desalted using Sep-Pak C18 cartridges (Waters, USA). The desalted peptides were then dried by vacuum centrifugation and used for glycopeptide enrichment.

**Yeast sample preparation**

The fission yeast, *Schizosaccharomyces pombe (S. pombe)* strains were kindly provided by Professor Meng-Qiu Dong from National Institute of Biological Sciences (Beijing). Washed fission yeast cells were snap-frozen in liquid nitrogen, ground to a fine powder with mortar and pestle in liquid nitrogen, and stored at -80 °C until use. The grinding powder from different organisms were processed to protein extraction and digestion. The powder was dissolved in fivefold-volume lysis buffer (4% SDS, 0.1 M Tris/HCl, pH 8.0) with protease inhibitor (1 mM PMSF, 1 mM cocktail), followed by boiling at 100 °C for 10 minutes, ultra-sonication for 5 minutes and centrifugation at 12,000 g at 18 °C for 30 minutes to collect protein extracts. The protein concentration was determined by BCA method. Proteins were then reduced in 10 mM dithiothreitol at 57 °C for 30 minutes, and then alkylated in dark by 20 mM iodoacetamide at room temperature for 30 minutes. After carbamidomethylation, six volumes of acetone were added to precipitate the proteins at −20 °C for at least 3 hours. The precipitates were dissolved in a denaturing buffer (8 M urea in 50 mM NH_4_HCO_3_) following a ten-fold dilution with 50 mM NH_4_HCO_3_. Then the proteins were digested into peptides by incubating with trypsin (a final enzyme-to-substrate ratio of 1:50) at 37 °C overnight, and then desalted using Sep-Pak C18 cartridges (Waters, USA). The desalted peptides were then dried by vacuum centrifugation and used for glycopeptide enrichment.

**Glycopeptide enrichment**

Glycopeptides were enriched by zwitterionic hydrophilic interaction liquid chromatography (ZIC-HILIC) method. Briefly, the desalted peptides of 1mg were resuspended in 300 μL loading buffer containing 80% ACN and 1% TFA and then loaded onto a homemade micro-column containing 50 mg of ZIC-HILIC particles (Merck Millipore, Darmstadt, Germany) packed onto a C8 disk. The flow-through was collected and reloaded onto the column for additional four times. Then, the column was washed with 200 μL loading buffer for four times, and finally eluted with 140 μL 0.1% TFA. The elution was collected and lyophilized.

**LC-MS/MS analysis**

LC-MS/MS analysis with triplicates for each sample were performed using a nanospray LC-MS/MS on an Orbitrap Fusion Tribrid system (Thermo Fisher Scientiﬁc, Waltham, MA, USA) ﬁtted with an EASY-nLC TM1100 system (Thermo Fisher Scientiﬁc, Waltham, MA, USA) that included a reverse-phase analytical column without the trap column. Solvent A was a 0.1% formic acid aqueous solution. Solvent B was acetonitrile containing 0.1% formic acid. The gradient lasted 2 hours in total: 2% to 40% in 105 minutes, an increase to 90% B in 3 minutes, hold 7 minutes with 90% B, then return to 2% B in 10 seconds and hold for another 4 minutes and 50 seconds. The samples were analyzed by SCE-HCD-MS/MS. The parameters for intact glycopeptide analysis were: (1) MS: scan range (m/z) = 350–2000; resolution = 120,000; included charge state = 2–6; dynamic exclusion after n times, n = 1; dynamic exclusion duration = 15 s; the precursors are selected under the “top speed” mode in Thermo Fusion mass spectrometer and each selected precursor was subjected to one HCD-MS/MS; (2) HCD-MS/MS: isolation window = 2; detector type = Orbitrap; resolution = 15,000; maximum injection time = 250 ms; collision energy = 30%; and stepped collision mode on with an energy difference of ±10% (10% as absolute value in the Orbitrap Fusion).

# Supplementary Note 2 Experimental methods for the fold change-(de)glycoproteome data obtaining.

**Sample preparation**

Human serum and fission yeast samples were prepared following the same procedure as that in Supplementary Note 1. Standard glycoprotein IgG were dissolved in 50 mM NH_4_HCO_3_ and then treated as same as that for human serum preparation.

**Glycopeptide enrichment**

Glycopeptides were enriched from each sample using the ZIC-HILIC method as that described in Supplementary Note 1. Then, glycopeptides were divided into four portions, two portions of 1 μg and two portions of 200 ng, among which, a portion of 1 μg and 200 ng glycopeptides were treated with PNGase F.

**PNGase F treatment**

For preparation of deglycopeptides, glycopeptide samples were dissolved in 100 mM NH_4_HCO_3_ buffer, added with PNGase F, and incubated at 37 °C overnight to remove N-glycans. After that, deglycopeptides were desalted, lyophilized, and stored at −80 °C for subsequent analysis.

**LC-MS/MS analysis**

All LC-MS/MS analyses were performed on a nanospray LC-MS/MS on an Orbitrap Fusion Tribrid system (Thermo Fisher Scientiﬁc, Waltham, MA, USA) ﬁtted with an EASY-nLC TM1100 system (Thermo Fisher Scientiﬁc, Waltham, MA, USA) that included a reverse-phase analytical column without the trap column. Solvent A was a 0.1% formic acid aqueous solution. Solvent B was acetonitrile containing 0.1% formic acid.

For glycopeptide analysis, the gradient lasted 2 hours in total: 2% to 40% in 105 minutes, an increase to 90% B in 3 minutes, hold 7 minutes with 90% B, then return to 2% B in 10 seconds and hold for another 4 minutes and 50 seconds. The samples were analyzed by SCE-HCD-MS/MS. The parameters for intact glycopeptide analysis were: (1) MS: scan range (m/z) = 350–2000; resolution = 120,000; included charge state = 2–6; dynamic exclusion after n times, n = 1; dynamic exclusion duration = 15 s; the precursors are selected under the “top speed” mode in Thermo Fusion mass spectrometer and each selected precursor was subjected to one HCD-MS/MS; (2) HCD-MS/MS: isolation window = 4; detector type = Orbitrap; resolution = 15,000; maximum injection time = 250 ms; collision energy = 30%; and stepped collision mode on with an energy difference of ±10% (10% as absolute value in the Orbitrap Fusion).

For deglycopeptide analysis, the gradient lasted 2 hours in total: 4% to 35% in 100 minutes, an increase to 65% B in 10 minutes, and another increase to 100% B in 2 minutes, then hold for another 8 minutes. The samples were analyzed by HCD-MS/MS. The parameters for deglycopeptide analysis were: (1) MS: scan range (m/z) = 350–1600; resolution = 120,000; included charge state = 2–4; dynamic exclusion after n times, n = 1; dynamic exclusion duration = 18 s; the precursors are selected under the “top speed” mode in Thermo Fusion mass spectrometer and each selected precursor was subjected to one HCD-MS/MS; (2) HCD-MS/MS: isolation window = 1.6; detector type = Orbitrap; resolution = 15,000; maximum injection time = 100 ms; collision energy = 28%.

# Supplementary Note 3 Sample preparation and LC-MS/MS Methods for the three benchmark datasets and HCC cell lines.

- 1. **Sample preparation for SILAC-labeled 293T cells**

**Cell culture and labeling**

The K0R0 medium containing Dulbecco’s modified Eagle’s medium (DMEM, HyClone, Logan, UT, USA) supplemented with 10% dialyzed fetal bovine serum (FBS, Gibco, Grand Island, NY, USA) was prepared as light medium for light labeling. The K6R6 medium containing DMEM with L-lysine (^13^C_6_, 99%) and L-arginine (^13^C_6_, 99%) and 10% dialyzed FBS was prepared as heavy medium (K6R6-medium) for heavy labeling.

Split a dish of 293T cells into two separate dishes, each containing K0R0 medium and K6R6 medium respectively. The cells were cultured at 37 °C in a humidified 5% CO_2_ incubator. When the cells are >80% confluent, rinse the cells with PBS, detach the cells with trypsin/EDTA and split the cells onto new dishes. Cell lines were grown for eight cell divisions in either K0R0 medium or K6R6 medium.

**Cell collection and protein extraction**

After SILAC labeling is complete, remove medium from the dishes, wash twice with ice cold PBS, and then completely remove PBS. The cells were collected in a tube and lysed in five-volume lysis buffer (4% SDS, 0.1M Tris/HCl, pH 8.0) with protease inhibitor (1 mM PMSF, 1 mM cocktail), followed by boiling at 100 °C for 10 minutes, ultrasonication for 20 minutes and centrifugation at 12,000 g at 18 °C for 30 minutes to collect protein extracts. The protein concentration was determined by BCA method.

**Protein digestion**

The proteins extracted from K0R0-labeled cells and K6R6-labeled cells were mixed with 1:1. Then, the protein mixtures were reduced in 10 mM dithiothreitol at 37 °C for 1 hour, and then alkylated in dark by 20 mM iodoacetamide at room temperature for 30 minutes. After that, six volumes of precooled acetone were added to precipitate the proteins at −20 °C for at least 3 hours. The precipitates were dissolved in a denaturing buffer (8 M urea in 50 mM NH_4_HCO_3_) following a ten-fold dilution with 50 mM NH_4_HCO_3_. Trypsin was added to a final enzyme-to-substrate ratio of 1:50 (w:w) and incubated at 37 °C overnight. The reactions were terminated by adding trifluoroacetic acid with final concentration of 0.5%. Finally, all digests were centrifuged at 16,000 g for 10 min and the supernatants were desalted using Sep-Pak C18 cartridges (Waters, USA). The desalted peptides were then lyophilized for subsequent enrichment procedures.

- 1. **Sample preparation for label-free HeLa cells**

HeLa cells were cultured in DMEM supplemented with 10% dialyzed FBS at 37 °C in a humidified 5% CO_2_ incubator. The cells were collected in a tube, and washed twice with ice cold PBS. Proteins were then extracted from cells, digested and desalted following the same procedures in sample preparation for SILAC-labeled 293T cells. The desalted peptides were lyophilized and stored at -80 °C for subsequent enrichment procedures.

- 1. **Sample preparation for TMT-labeled 293T cells**

293T cells were cultured in DMEM supplemented with 10% dialyzed FBS at 37 °C in a humidified 5% CO_2_ incubator. The cells were collected in a tube, and washed twice with ice cold PBS. Proteins were then extracted from cells, digested and desalted following the same procedures in sample preparation for SILAC-labeled 293T cells. The desalted peptides were lyophilized and stored at -80 °C for subsequent TMT-labeling procedure.

The digests were divided into two aliquots, each of which were labeled with the TMT6plex^TM^ label reagents TMT^6^-128 and TMT^6^-131 following the TMT6plex^TM^ isobaric label reagent product manual (Thermo Fisher Scientiﬁc, Waltham, MA, U.S.A.). Briefly, per 100 μg protein digests were resuspended with 100 μL of 100 mM TEAB, pH 8.5. A total of 41 μL of anhydrous acetonitrile was added into each 0.8 mg vials and allowed to dissolve for 5 minutes with occasional vortex. After briefly centrifugation the tube, the 41 μL of the reagent solution was added to each 100 μL sample (containing 100 μg protein digests) and incubated for 1 hour at room temperature. Then, 8 μL of 5% hydroxylamine was added to the sample and incubated for 15 minutes to quench the reaction. Equal amounts of each samples were combined in a new microcentrifuge tube and lyophilized. Then the samples were cleaned up using Sep-Pak C18 cartridges (Waters, USA) and lyophilized for subsequent enrichment procedures

- 1. **Sample preparation for HCC cell lines**

Three hepatocellular carcinoma cell lines, Hep3B, MHCC97L and MHCCLM3, were adopted for SILAC labeling. The K0R0 medium containing DMEM supplemented with 10% dialyzed FBS was used as light medium for labeling of 97L. The K4R6 medium containing DMEM with L-lysine (D_4_, 99%) and L-arginine (^13^C_6_, 99%) supplemented with 10% dialyzed FBS was used as median medium for labeling of Hep3B. The K8R10 medium containing DMEM with L-lysine (^13^C_6_, 99%; ^15^N_2_, 99%) and L-arginine (^13^C_6_, 99%; ^15^N_4_, 99%) supplemented with 10% dialyzed FBS was used as heavy medium for labeling of LM3. Hep3B, MHCC97L and MHCCLM3 cells were cultured in the above medium respectively following the same experimental procedures with that for SILAC-293T cells. After complete SILAC labeling, the three kind of cells were collected followed by protein digestion as that for 293T cells, respectively. Then the proteins extracted from the three cell lines were mixed with 1:1:1, digested, desalted using Sep-Pak C18 cartridges, and lyophilized for subsequent processing.

The digests were fractionated by hydrophilic interaction liquid chromatography (HILIC) using a Waters UPLC system coupled with a HILIC column (Welch, Ultimate HILIC Amide, 4.6× 250 mm, 5 μm). The digests were re-dissolved in phase A (10 mmol/L ammonium formate, H_2_O, pH 4.5 adjusted with FA). The gradient was as follows: 100% B (75% ACN and 10 mmol/L ammonium formate) for 5 min, 100–90% B in 3 min, 90–40% B in 56 min, 40–20% B in 3 min, return 100% B in 0.1 min, and hold until the end of gradient. The flow rate was 1 mL/min and the column temperature maintained at 45 °C. The collection started at the 4^th^ minute to the 70^th^ minute with 1 minutes in turn and were combined as the following conditions: fraction 1 (1-11), fraction 2 (12-19), fraction 3 (20-27), fraction 4 (28-35), fraction 5 (36-43), fraction 6 (44-51), and fraction 8 (52-59), and fraction 9 (60-67). Then, each of fractions was desalted and divided into two parts. One part is used for LC-MS/MS analysis for proteome identification and quantitation. The other part is used for glycopeptide enrichment and LC-MS/MS analysis for intact glycopeptide identification and quantitation.

- 1. **Glycopeptide enrichment**

Glycopeptides were enriched by zwitterionic hydrophilic interaction liquid chromatography (ZIC-HILIC) method. Briefly, the desalted peptides of 500 μg - 1 mg were resuspended in 300 μL loading buffer containing 80% ACN and 1% TFA and then loaded onto a homemade micro-column containing 50 mg of ZIC-HILIC particles (Merck Millipore, Darmstadt, Germany) packed onto a C8 disk. The flow through was collected and reloaded onto the column for additional four times. Then, the column was washed with 200 μL loading buffer for four times, and finally eluted with 140 μL 0.1% TFA. The elution was collected and lyophilized.

- 1. **LC-MS/MS analysis**

All LC-MS/MS analyses were performed on LC-MS/MS on an Orbitrap Fusion Tribrid system (Thermo Fisher Scientiﬁc, Waltham, MA, USA) equipped with an EASY-nLC TM1100 system (Thermo Fisher Scientiﬁc, Waltham, MA, USA) that included a reverse-phase analytical column without the trap column. Solvent A was a 0.1% FA aqueous solution. Solvent B was ACN containing 0.1% FA. Detailed LC-MS/MS parameters are listed below.

|  | | SILAC-labeled 293T cell | label-free HeLa cell | TMT-labeled 293T cell | SILAC-labeled HCC cell lines for intact glycopeptide | SILAC-labeled HCC cell lines for proteome |
| --- | --- | --- | --- | --- | --- | --- |
| LC | Column | C18 column 50 cm×75 μm i.d. (Thermo) | | | | |
|  | Flow rate | 250 nl/min | 250 nl/min | 300 nl/min | 200 nl/min | 200 nl/min |
|  | total gradient time | 360 min | 360 min | 120 min | 240 min | 120 min |
|  | Elute gradient | 1–30% B in 330 min, 30–45% B in 15 min, 45-90% in 1 min, 90% for 7 min, 90%-1% in 10 s, 1% for 6'50 s | 1–30% B in 330 min, 30–45% B in 15 min, 45-90% in 1 min, 90% for 7 min, 90%-1% in 10 s, 1% for 6'50 s | 2–40% B in 105 min, 40–90% B in 3 min, 90% for 7 min, 90%-2% in 10 s, 2% for 4'50 s | 1–20% B in 180 min, 20–30% B in 42 min, 30-90% in 3 min, 90% for 7 min, 90%-1% in 10 s, 1% for 7'50 s | 3–8% B in 4 min, 8–24% B in 80 min, 24-35% in 24 min, 35-90% in 2 min, 90% for 4 min, 90%-3% in 10 s, 3% for 5'50 s |
| MS1 | Scan range (m/z) | 350-2000 | 350-2000 | 350-2000 | 350-2000 | 350-1550 |
|  | Resolution | 120,000 | | | | 240,000 |
|  | Included charge state | 2-6 | | | | |
|  | dynamic exclusion after n times | 1 | | | | 1 |
|  | dynamic exclusion duration | 15 s | | | | 60 s |
|  | Data acquisition mode | DDA | | | | |
| MS/MS | Resolution | 15,000 | | | | 30,000 |
|  | Maximum injection time | 250 ms | 250 ms | 250 ms | 250 ms | 64 ms |
|  | Collision energy | HCD@30%±10% | | | | HCD@36% |

# Supplementary Note 4 Methods for invitro molecular biology experiments.

**Cell lines and cell culture.** Hepatocellular carcinoma MHCC97L and MHCCLM3 cell lines were cultured in Dulbecco’s modified Eagle’s medium (DMEM, HyClone, Logan, UT, USA) supplemented with 10% fetal bovine serum (FBS, Gibco, Grand Island, NY, USA) and 10 U/mL penicillin streptomycin (Gibco by Invitrogen, Carlsbad, CA, USA) at 37 °C in a humidified 5% CO2 incubator.

**Western blot assay.** The cell lysate was collected and prepared in ice-cold RIPA lysis and extraction Buffer (Thermo Fisher Scientific, Waltham, MA, USA), supplemented with a complete protease inhibitor mixture (Roche Diagnostics, Penzberg, Germany). Total cellular proteins were resolved by SDS-PAGE and transferred to a polyvinylidene difluoride (PVDF) membrane. After blocking nonspecific binding with TBS/T (0.1%) containing 5% non-fat milk for 1 h at room temperature, the membrane was incubated with the following different primary antibodies: anti-FUT8 (66118-1-lg, 1:1000), anti-FLAG (80010-1-RR, 1:5000), anti-β-Actin (66009-1-lg, 1:10000) (Proteintech, Rosemont, IL, USA), anti-L1CAM (ab182407, 1:1000), anti-L1CAM (ab24345, 1:1000) and anti-β-Tubulin (ab151318, 1:1000) (Abcam, Cambridge, UK). The membrane was washed with TBS/T four times to remove the unbound antibody and then incubated with the secondary antibody (HRP-conjugated goat anti-mouse IgG or goat anti-rabbit IgG, 1: 10000; Affinity Bioscience, Jiangsu, China) for 1 h at room temperature. Protein bands were visualized with an ECL kit (BeyoECL Plus, Beyotime Institute of Biotechnology, Shanghai, China).

**Lectin enrichment** **and** **immunoblot assay.** A total of 1000 μg of cell lysate was mixed with 320 μL of agarose bound Pisum Sativum Agglutinin (PSA) lectin and agarose bound Lens Culinaris Agglutinin (LCA) lectin (Vector laboratories, Burlingame, CA, USA) in 650 μL binding buffer containing 2 mM MnCl_2_, 2 mM CaCl_2_, and 1 mM NaCl_2_ and incubated with rotation at 4 °C overnight, for specific enrichment of core-fucosylated glycorpteins. Then, the beads were washed three times with binding buffer and subsequently extracted with SDS-PAGE sample buffer at 99 °C for 10 min. The samples were separated by 4-20% Bis-Tris-PAGE and subjected to immunoblotting with antibody anti-L1CAM (ab182407, 1:1000). For lectin blot, after separation through Bis-Tris—PAGE, the samples were transferred to PVDF membranes. The membrane was washed with TBS/T four times to remove the unbound antibody and then incubated with the secondary antibody (HRP-conjugated goat anti-mouse IgG or goat anti-rabbit IgG, 1: 10000; Affinity Bioscience, Jiangsu, China) for 1 h at room temperature. Protein bands were visualized with an ECL kit (BeyoECL Plus, Beyotime Institute of Biotechnology, Shanghai, China).

**Wound healing assay.** Cells were seeded into a 24-well culture plate with the number of 1 × 10^5^ cells/well. When the cell density reached 90% confluency, a cell monolayer was scratched gently with a sterile pipette tip. After washing with PBS twice, fresh medium was added. Cell migration was observed under a microscope (DMi8, Leica, Wetzlar, Germany) and imaging was performed at different time points.

**Transwell migration assay.** Transwell assay was conducted using a transwell chamber (Corning, NY, USA) with an aperture of 8 μm to estimate cell migration. A total of 5 × 10^4^ cells was suspended in 100 μL serum-free medium and then added into the upper chamber, whereas 600 μL of 10% FBS medium was added in the lower chamber. After incubation at 37 °C with 5% CO_2_ for 36 h, the non-migrating cells on the upper chamber were removed with cotton swabs, and the migrated cells were fixed with methanol and stained with 0.1% crystal violet. Five fields were randomly selected and the number of migrated cells was counted under the microscope.

**Matrigel invasion assay.** As for the transwell invasion assay, the upper chamber membranes were coated with matrigel (Corning, NY, USA). 1 × 10^5^ cells were added into the upper chamber in serum-free medium, the rest was the same as the migration assay.

**siRNA and plasmid transfection.** FUT8-specific siRNAs (sequence: 5′-GUGGAGUGAUCCUGGAUAUTT-3′, 5′-AUAUCCAGGAUCACUCCACTT-3′), L1CAM-specific siRNAs (sequence: 5′-GUGGAGUGAUCCUGGAUAUTT-3′, 5′-AUAUCCAGGAUCACUCCACTT-3′) and negative control siRNA (sequence: 5′-UUCUCCGAACGUGUCACGUTT-3′, 5′-ACGUGACACGUUCGGAGAATT-3′) were purchased from GenePharma (Shanghai, China). The pCMV3-entry (empty)vector and pCMV3-L1CAM-FLAG vector (with a FLAG tag at C-terminal) were purchased from Sino Biological (Beijing, China). The transfection of siRNAs and plasmid was performed using Lipofectamine 3000 kit (Invitrogen, Carlsbad, CA, USA) according to the manufacturer’s protocol.

**Plasmid site-directed mutagenesis.** A mammalian expression pCMV3 vector containing the human L1CAM cDNA with a FLAG tag at C-terminal (pL1CAM-FLAG) was obtained from Sino Biological (Beijing, China). pL1CAM-FLAG plasmid was used as template to generate the pL1CAM (N979Q)-FLAG mutant by site-directed mutagenesis method. In brief, using the template-specific mutagenic primers (forward: 5’-CGAACTTCGGACACACCAGCTGACCGATCTCAGCC-3’, reverse: 5’-GGCTGAGATCGGTCAGCTGGTGTGTCCGAAGTTCG-3’), the pL1CAM-FLAG plasmid was amplified by PCR with Phanta DNA polymerase (Vazyme, P501). The PCR products were digested with DpnI restriction enzyme, transformed into TOP10 competent cells, and the positive clones were picked up to confirm the corrected pL1CAM (N979Q)-FLAG mutant by DNA sequencing.
