# Supplementary material for: pGlycoQuant with a deep residual network for quantitative glycoproteomics at intact glycopeptide level
Source: Nat Commun. 2022 Dec 7;13:7539. doi: 10.1038/s41467-022-35172-x (PMC9729625; doi:10.1038/s41467-022-35172-x)
Supplement: Supplementary file 3 — Description of Additional Supplementary files [file 41467_2022_35172_MOESM3_ESM.pdf]

Description of Additional Supplementary Files

File Name: Supplementary Data 1
Description: The glycopeptide spectra quantified from Human IgG by MIR.

File Name: Supplementary Data 2
Description: The quantitative results of the proteome in three different metastatic HCC cell lines.

File Name: Supplementary Data 3
Description: The quantitative results of the N-glycoproteome in three different metastatic HCC cell lines.

File Name: Supplementary Data 4
Description: The comparison results of the differential proteins between the three different metastatic HCC cell lines.

File Name: Supplementary Data 5
Description: The comparison results of the differential site-specific glycans between the three different metastatic HCC cell lines.

File Name: Supplementary Data 6
Description: The information of the glycan-related genes in proteomic quantitative results in three HCC cell lines.

File Name: Supplementary Data 7
Description: The comparison results of the differential site-specific glycans without and with normalization to protein abundance between the three different metastatic HCC cell lines.
